# Identification of New Lupane-Type Triterpenoids as Inverse Agonists of RAR-Related Orphan Receptor Gamma (RORγ)

**DOI:** 10.1021/acs.jnatprod.5c00416

**Published:** 2025-07-28

**Authors:** Patrik F. Schwarz, Alexander F. Perhal, Famke Guder, Jorge Enrique Hernández González, Kerrin Janssen, Ece Sağıroğlu, Ammar Tahir, Johannes Kirchmair, Natacha Rochel, Verena M. Dirsch, Ya Chen

**Affiliations:** † Department of Pharmaceutical Sciences, Division of Pharmacognosy, Faculty of Life Sciences, 27258 University of Vienna, Josef-Holaubek-Platz 2, 1090 Vienna, Austria; ‡ Vienna Doctoral School of Pharmaceutical, Nutritional and Sport Sciences (PhaNuSpo), 27258 University of Vienna, 1090 Vienna, Austria; § Institute of Genetics and Molecular and Cellular Biology, 27083 University of Strasbourg, CNRS UMR7104, INSERM U 1258, Illkirch-Graffenstaden, 67404, France; ∥ Department of Pharmaceutical Sciences, Division of Pharmaceutical Chemistry, Faculty of Life Sciences, 27258 University of Vienna, Josef-Holaubek-Platz 2, 1090 Vienna, Austria; ⊥ Department of Physics, Sao Paulo State University, Rua Cristóvão Colombo 2265, São José do Rio Preto, CEP 15054-000, Brazil; # Institute of Physical and Theoretical Chemistry, 26527 Technische Universität Braunschweig, Gaußstraße 17, 38106 Braunschweig, Germany

## Abstract

Targeting retinoic acid-related orphan receptor γ (RORγ) with inverse agonists presents a promising therapeutic strategy for treating autoimmune diseases, including psoriasis, rheumatoid arthritis, and multiple sclerosis. Through structure-based virtual screening, we identified a lupane-type pentacyclic triterpenoid, (2Z)-2-(2-furanylmethylene)-3-oxolup-20(29)-en-28-oic acid (**15**), as a new inverse agonist of RORγ. The compound exhibited IC_50_ values of 0.4 μM and 0.9 μM in Gal4-RORγ and full-length RORγ luciferase assays, respectively. Compound **15** showed improved potency and efficacy compared to a structurally related known inverse agonist, betulinic acid. Among the four additional analogues tested (**15.1**-**15.4**), two (**15.2** and **15.3**) also demonstrated RORγ inverse agonist activity with low micromolar IC_50_ values in Gal4-RORγ luciferase assay. Real-time quantitative polymerase chain reaction experiments confirmed that compounds **15**, **15.2**, and **15.3** downregulated RORγ target genes. Thermal shift assays showed that both betulinic acid and **15** stabilized the RORγ ligand-binding domain. Molecular docking and structure–activity relationship analysis revealed distinct binding modes within the RORγ ligand-binding domains, further supported by site-directed mutagenesis. These findings expand the repertoire of RORγ inverse agonists based on the pentacyclic triterpenoid scaffolds.

## Graphical abstract

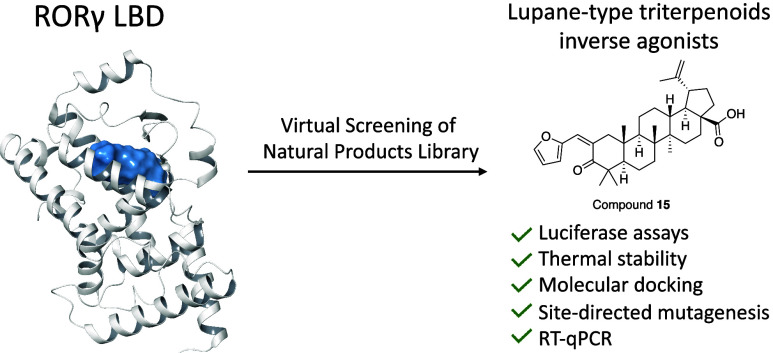

Retinoic acid-related orphan receptors (RORs) are a subfamily of nuclear receptors that regulate important physiological processes related to metabolism and the immune system. RORs bind to ROR response elements (RORE) as monomers through their DNA-binding domain (DBD). Like other nuclear receptors, RORs function as transcription factors that can be influenced by small molecules binding to their ligand-binding domains (LBD).^1^

The three members of the ROR subfamily are RORα,^2^ RORβ,^3^ and RORγ.^4^ Various isoforms of these proteins exist, produced by differential promoter usage or exon splicing.^5^ RORα is expressed in various tissues within the body (e.g., central nervous system, liver, thymus, skeletal muscle, and adipose tissue).^5–7^ Its deletion shows beneficial effects (e.g., in glucose and lipid metabolism^8–10^ ) but also detrimental impacts (e.g., by causing cerebral and muscular atrophy and immunodeficiencies).^11^ RORβ is predominantly expressed in the central nervous system, specifically in regions associated with processing sensory information and regulating the circadian rhythm.^5,12,13^ Hence, RORβ knockout mice experience blindness in adulthood and abnormal circadian behavior.^12,14^ While RORγ (like RORα) is expressed in various tissues like the liver (often coexpressed with RORα),^5^ where it is involved in hepatic gluconeogenesis,^15^ its isoform RORγt is exclusively expressed in the thymus.^4,16^ As RORγ and RORγt (summarized as RORγ in this study) differ solely in their N termini but not their LBD, selective pharmacological modulation of only one isoform is not feasible, at least via the LBD.^17^

RORγ is a potential drug target for numerous autoimmune diseases^18,19^ because it is a crucial transcription factor in the differentiation of naive CD4^+^ T cells into pro-inflammatory T helper 17 (Th17) cells.^20^ Th17 cells are known as host defenders but are also involved in autoimmune diseases such as psoriasis, rheumatoid arthritis, and multiple sclerosis, through the release of their main cytokine, interleukin-17 (IL-17).^21,22^ While monoclonal antibodies against IL-17 are successfully used for treating psoriasis and related diseases,^23^ they showed a low efficacy or detrimental effects in other Th17-mediated diseases, such as rheumatoid arthritis or inflammatory bowel disease.^24^ In part, this paradox can be explained by a change of the Th17 phenotype, from predominantly producing IL-17 to predominantly producing interferon-γ (IFN-γ) in the course of some diseases.^24^ In these cases, monoclonal antibodies against IL-17 are ineffective^25^ and other therapeutic options, such as RORγ inhibitors, could fill the ensuing treatment gap by inhibiting Th17 differentiation rather than the cytokines these cells produce. RORγ inhibitors can be categorized as inverse agonists or antagonists based on whether RORγ is considered unliganded (inverse agonist) or liganded (antagonist) inside the cell.^26^ As it was shown that apo-RORγ can adopt an active conformation,^27^ inhibitory compounds will be referred to as inverse agonists in this study.

Examples of RORγ inverse agonists include synthetic compounds (e.g., SR2211,^28^ IMU-935,^29^ and JNJ-61803534 ^30^) and natural products (e.g., digoxin,^31^ ursolic acid,^32^ diosgenin,^33^ CYP11A1-derived hydroxyvitamin D derivatives,^34^ and hydroxylumisterols,^35^ reviewed in ref 36). Such compounds were successfully used in several *in vivo* models to treat autoimmune diseases linked to Th17 cells.^32,37–39^ RORγ agonists, on the other hand, could prove useful as cancer therapeutics,^40,41^ with cintirorgon even entering clinical trials.^42^ While the development of RORγ modulators has faced challenges, partly due to their poor physicochemical properties, limited selectivity, safety concerns, and the limited understanding of the endogenous ligand(s),^1,19,43,44^ recent clinical trials with the potent RORγ inverse agonists IMU-935 and JNJ-61803534 unveiled a favorable safety profile for both of these synthetic compounds.^29,30^ The same is true for the aforementioned RORγ agonist cintirorgon.^42^

Computational approaches have played a crucial role in discovering, designing, and optimizing RORγ modulators. For example, Rauhamäki et al.^45^ employed Glide,^46–48^ a well-established docking algorithm, and PANTHER,^49^ a rapid algorithm for assessing the compatibility of the ligand binding site and small organic compounds concerning shape and electrostatic properties, to screen a library of commercially available screening compounds for potential inverse agonists of RORγ. Eleven of the 34 purchased and tested compounds were confirmed to be inverse agonists, with the most potent compound having an IC_50_ of 590 nM.

Moret et al.^50^ developed a generative deep learning approach which they employed to generate molecular structures of potential inverse agonists of RORγ. They synthesized the three most promising designs, which they confirmed in Gal4-RORγ hybrid reporter gene assays to act as inverse agonists of RORγ, with IC_50_ values in the low micromolar and submicromolar range.

Here, we report on the search for new natural products and natural product-like compounds that act as inverse agonists of RORγ. Natural products have undergone “nature’s own high-throughput screening through evolution” (Ian Paterson, in ref 51) and are thus often optimized to fulfill specific biological purposes, such as binding to certain proteins.^52^ They can also serve as templates for chemical modifications to address unfavorable inherent properties, such as poor pharmacokinetics. One example of this is the conversion from paclitaxel (a natural product) to docetaxel (a natural product derivative).^53^ Using a structure-based virtual screening approach, we selected 23 compounds from MolPort’s Database of Purchasable Natural Compounds,^54^ a library of more than 100,000 commercially available natural products and their derivatives, for experimental testing. One of the compounds, a lupane-type triterpenoid structurally related to betulinic acid (**15**, (2Z)-2-(2-furanylmethylene)-3-oxolup-20(29)-en-28-oic acid), was found to be an effective inverse agonist of RORγ using luciferase assays. Follow-up work identified three derivatives (i.e., **15.1**, **15.2,** and **15.3**) as RORγ inverse agonists. These compounds demonstrated varying capacities to suppress RORγ target gene expression and stabilize RORγ LBD in thermal stability assays. The structure–activity relationship (SAR) and binding modes of these compounds with RORγ-LBD were explored using molecular docking and site-directed mutagenesis.

## Results and Discussion

### Virtual Screening and Compound Selection

The 113,687 prepared compounds of MolPort’s Database of Purchasable Natural Compounds were docked with Glide^46–48^ against four X-ray structures of human RORγ (PDB IDs 4WQP,^55^ 5NTP,^56^ 5NU1,^56^ and 6J3N^57^). The four protein structures were selected from over 150 publicly available X-ray structures to work with a small set of high-quality structures representing the conformational space of the orthosteric ligand binding pocket. The selection was guided by evaluation of structural resolution and electron density maps, binding site flexibility, and ligand representativeness, as detailed in the Experimental Section.

From the four hit lists obtained from docking, 23 compounds were selected for purchasing and experimental testing (Table S1). The chosen compounds match the following criteria:

- **Compatibility:** The docking poses obtained during virtual screening must indicate the compatibility of the compounds’ pharmacophoric features and shapes with the ligand binding site.
- **Novelty:** No known modulators of RORγ are highly similar to any candidate compound (according to a similarity search with CAS SciFinder (Chemical Abstracts Service, Columbus, OH) on September sixth, 2022, using a similarity threshold of 80). The similarity search was based on 2D structural descriptors and Tanimoto coefficient calculations, as implemented by CAS SciFinder.
- **Diversity:** The candidate compounds are chemically diverse, meaning all selected compounds are based on distinct Murcko scaffolds^58^ (i.e., 23 compounds, 23 distinct Murcko scaffolds).
- **Purchasability and cost efficiency:** The candidate compounds must be available from MolPort in sufficient quantity (5 mg) at a moderate cost.

### Initial Biological Evaluation of the 23 Compounds Selected by Virtual Screening

The 23 compounds selected by virtual screening **(** Table S1) were tested for their ability to function as inverse agonists of RORγ in a Gal4-RORγ mammalian one-hybrid luciferase assay. All compounds were evaluated at a concentration of 3 μM. SR2211, a selective inverse agonist of RORγ,^28^ was used as a positive control at 1 μM concentration. Among the 23 tested compounds, **15** ((2Z)-2-(2-furanylmethylene)-3-oxolup-20(29)-en-28-oic acid) was identified as a new inverse agonist of RORγ (Figures 1 and 2). Interestingly, **5** exhibited agonistic activity at a concentration of 3 μM (Figure 1). However, since we focused on identifying and characterizing inverse agonists rather than agonists of RORγ, we did not pursue this compound further.

**Figure 1:**
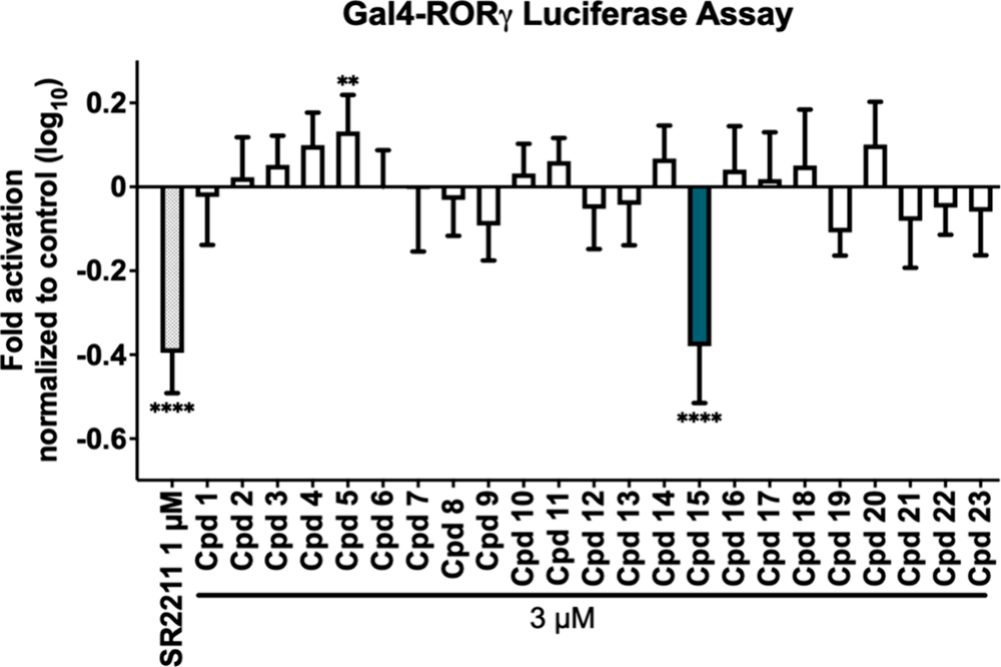
Experimental evaluation of the 23 candidate modulators of RORγ, selected by virtual screening from MolPort’s Database of Purchasable Natural Compounds. Compounds **1**-**23** were tested in a cell-based Gal4-RORγ luciferase assay at 3 μM for their inverse agonistic activities on RORγ. The published RORγ inverse agonist SR2211 was used at 1 μM as a positive control. The luminescence signals derived from the luciferase reporter were normalized to eGFP fluorescence and expressed as fold activation normalized to the vehicle control. Bar charts represent transactivation activities as mean ± SD of three biological replicates (*n* = 3) measured in technical quadruplicates. One-way ANOVA, followed by Dunnett’s post hoc test, was used for statistical analysis. ****p ≤ 0.0001, ***p ≤ 0.001, *p ≤ 0.05, no indication *p* > 0.05 as compared to vehicle control.

**Figure 2:**
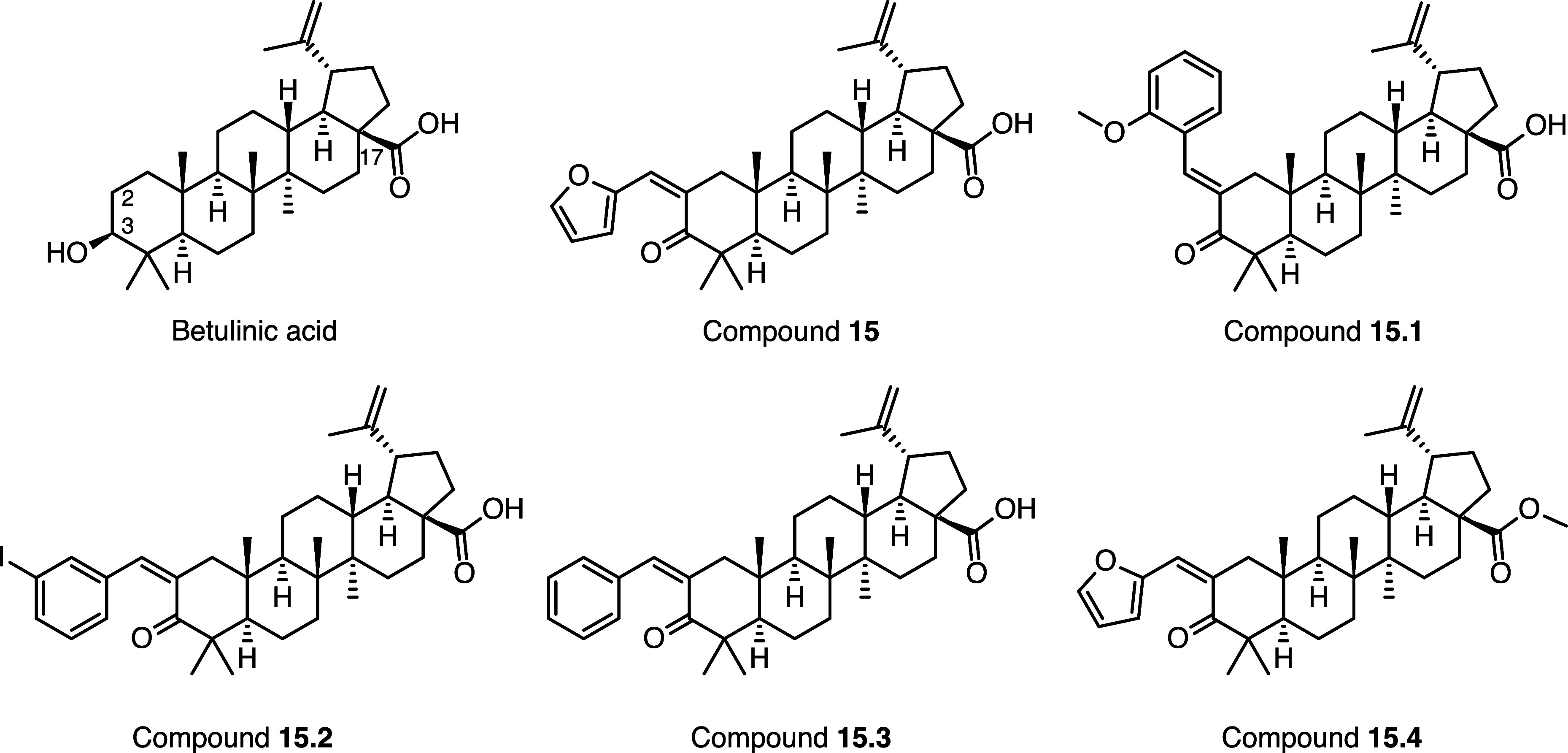
Chemical structures of betulinic acid, **15**, and the follow-up derivatives, **15.1** to **15.4**.

Compound **15** showed potent, concentration-dependent, inverse agonistic activity on RORγ: We determined an *I*_max(%)_ value of 76% (5 μM) and an IC_50_ value of 0.4 μM (Figure 3A and Table 1). The compound’s potency in the Gal4-RORγ assay is comparable to that of published values for the synthetic inverse agonist SR2211.^28^

**Figure 3:**
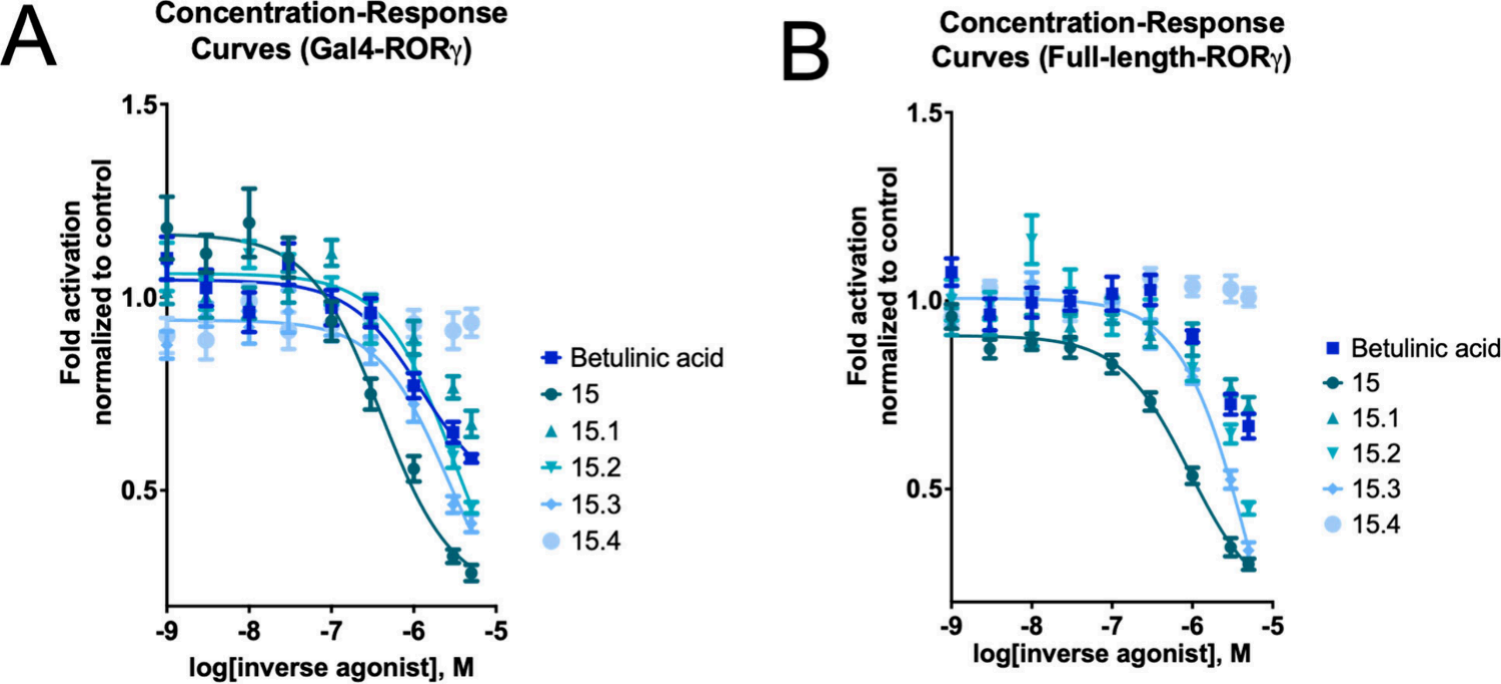
Concentration-dependent activity of betulinic acid and a series of lupane-type triterpenoids against the nuclear receptor RORγ. The panels display concentration–response data for the indicated compounds in (A) a Gal4-RORγ reporter assay and (B) a full-length RORγ assay. Data were analyzed by three-parameter logistic nonlinear regression (Hill slope constrained to −1.0). A fitted curve is displayed, and an IC_50_ value was determined only for compounds yielding a statistically reliable fit. A fit was considered reliable if it met quantitative criteria: R^2^ > 0.5 and a 95% confidence interval of the IC_50_ spanning less than a 10-fold range. For compounds that failed these statistical criteria or showed no clear concentration–response trend upon visual inspection, data points are shown without a curve. Due to cytotoxicity, compounds were not tested at concentrations above 5 μM. All data points represent the mean ± SEM of at least three biological replicates (*n* ≥ 3), each measured in technical quadruplicates.

**Table 1:** Measured and Computed Properties for Betulinic Acid and Other Lupane-Type Triterpenoids

	Gal4-RORγ luciferase assay		Full-length RORγ luciferase assay				
	IC_50_ (μM)	*I*_max(%)_ at 5 μM	IC_50_ (μM)	*I*_max(%)_ at 5 μM	ΔTm measured with nano differential scanning fluorimetry (mean ± SD, °C)^a^	Purity measured with liquid chromatography–mass spectrometry (%)	Glide docking score with PDB structure 6J3N (kcal/mol)^b^
Betulinic acid	1.2^c^	47	n.d.^d^	38	9.1 ± 0.2	≥98.0^e^	–9.8
**15**	0.4^f^	76	0.9^g^	69	9.6 ± 0.4	90.5	–12.3
**15.1**	n.d.^d^	34	n.d.^d^	24	5.9 ± 0.4	95.0	n.a.^h^
**15.2**	3.1^i^	58	n.d.^d^	55	2.2 ± 0.5	93.5	–13.4
**15.3**	2.7^j^	53	>5.0^k^	67	2.8 ± 0.2	94.8	–13.1
**15.4**	n.d.^l^	0	n.d.^l^	0	1.1 ± 0.2	95.8	–11.4

a Thermal shift represents the difference in the melting temperature (Tm) of RORγ-LBD in the presence and absence of a ligand.

b The best docking scores obtained with Glide as part of the binding mode studies. Note that the docking scores reported in this table differ slightly from those reported in Table S1 because of minor differences in the docking protocol and the use of different versions of Glide (see the Experimental Section for details).

c 95% confidence interval: 0.5–3.2 μM, R^2^ = 0.6.

d An IC_50_ value was not determined if the model fit failed to meet key statistical criteria (R^2^ < 0.5 or a 95% confidence interval of the IC_50_ > 10-fold), or if the data showed no clear concentration–response trend upon visual inspection.

e Purity data provided by the vendor (Sigma-Aldrich; B8936).

f 95% confidence interval: 0.2–0.7 μM, R^2^ = 0.8.

g 95% confidence interval: 0.6–1.3 μM, R^2^ = 0.9.

h No docking pose due to a different double bond configuration.

i 95% confidence interval: 1.2–7.8 μM, R^2^ = 0.7.

j 95% confidence interval:1.1–6.6 μM, R^2^ = 0.6.

k Determined IC_50_ value exceeds the highest concentration tested and is therefore only an estimate. Estimated IC_50_ = 5.7 μM (95% confidence interval:2.6–12.4 μM, R^2^ = 0.9).

l An IC_50_ value could not be determined due to a complete lack of inhibition.

Additional verification of the suppression of RORE-dependent transcriptional activity was obtained with an assay utilizing full-length RORγ and a luciferase reporter regulated by RORE. For **15**, the IC_50_ value measured with this assay was 0.9 μM, and the *I*_max(%)_ value was 69% at a concentration of 5 μM (Figure 3B and Table 1), thus confirming the observations made in the Gal4 assay.

The possibility that the observed effects were due to cytotoxicity was excluded using a resazurin conversion assay. As shown in Figure S1A, no cytotoxic effects of **15** were observed at a concentration of 5 μM in HEK293 cells. Furthermore, **15** did not inhibit luciferase enzyme activity at 5 μM (see the Experimental Section for details).

### Molecular and Biological Properties of Compound 15 and Related Triterpenoids

Compound **15** was identified during structure-based virtual screening performed using an X-ray crystal structure of the human RORγ ligand-binding domain (RORγ-LBD) in complex with ursonic acid (PDB structure 6J3N;^57^ the bound ligand was removed for docking). Both **15** and ursonic acid are pentacyclic triterpenoids, which represent one of the most prolific ring systems in natural products.^59^ The MolPort Database of Purchasable Natural Compounds contains 7,477 terpenoids classed by pathway among all 101,626 compounds assigned at least one pathway level class identified by NPClassifier.^60^ NPClassifier is a deep learning tool trained on natural products with ontology classification labels that can automatically classify structures of natural products into pathways, superclasses, and classes.

In the MolPort Database of Purchasable Natural Compounds, terpenoids make up the third-largest superclass of natural products (8%) after alkaloids (60%) and shikimates and phenylpropanoids (25%). Among the 7,477 terpenoids, 3,116 are classified as triterpenoids or steroids. With PDB structure 6J3N, Glide successfully placed many triterpenoids and steroids in the ligand binding pocket and assigned good docking scores. More specifically, together with 126 other compounds, **15** was classified as a lupane triterpenoid by NPClassifier.

Compound **15** was ranked 16th among the 30,697 compounds successfully docked with Glide to PDB structure 6J3N (Glide docking score of −12.5 kJ/mol). In contrast, Glide did not produce docking poses for most triterpenoids and steroids, including **15**, with the other three X-ray structures of RORγ-LBD used in this virtual screening campaign (i.e., 4WQP, 5NTP, and 5NU1).

Compound **15** is structurally related to betulinic acid, another lupane-type triterpenoid and known inverse agonist of RORγ^61^ (Figure 2). While several triterpenoids and steroids have been known to act as inverse agonists or agonists of RORγ for some time (examples are shown in Figure 4), lupane-type triterpenoids were linked to RORγ inverse agonism only recently.^61^

**Figure 4:**
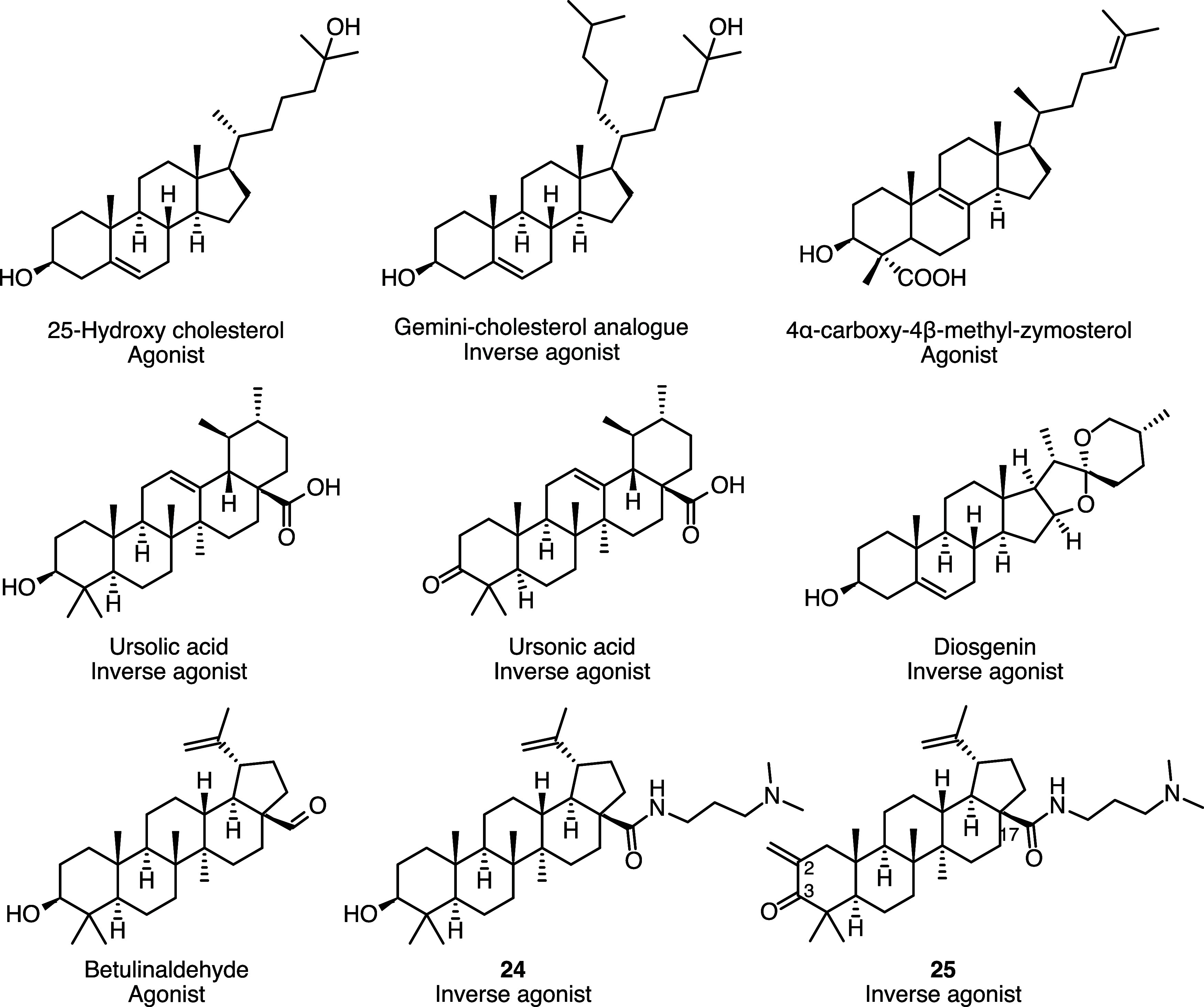
Representative triterpenoids and steroids as RORγ inverse agonists or agonists, collected from refs 33, 57, 61–65 .

Compared to betulinic acid, **15** is substituted with a furan-2-ylmethylidene moiety in position 2 and has a keto group instead of a hydroxyl group at position 3. Of note, substitution at position 2 of triterpenoid and steroid ligands of RORγ has so far been described only for one synthetic derivative of betulinic acid (**25**), which carries a methylene moiety at this position (Figure 4).

At 5 μM concentration, **15** showed a higher *I*_max(%)_ than betulinic acid in both the Gal4-RORγ luciferase assay (76% vs 47%) and the full-length RORγ luciferase assay (69% vs 38%) (Figure 3A and B, and Table 1). Both **15** and betulinic acid demonstrated no cytotoxic effects in the resazurin conversion assay at a concentration of 5 μM (Figure S1A). Taken together, these results indicate that **15** inhibits RORγ with relatively high potency and efficacy, while betulinic acid shows only moderate effects on RORγ.

### Hit Follow-up Studies on Compound 15

Four derivatives of **15** were purchased from MolPort for experimental testing (Figure 2, **15.1**, **15.2**, **15.3**, and **15.4**). These compounds were all ranked among the top 50 hits of the 30,697 compounds successfully docked to PDB structure 6J3N. However, they were deprioritized to increase the structural diversity of the compounds picked for initial experimental evaluation.

The four follow-up compounds differ from **15** by a single transformation: **15.1**, **15.2**, and **15.3** have the furan ring present in **15** replaced by a 2-methoxyphenyl, 3-iodophenyl, and phenyl moiety, respectively. Compound **15.4** has the carboxylic acid in **15** replaced by a methyl ester moiety. Compound **15.1** also differs in the configuration of the double bond in position 2 (E) compared to other derivatives, which, according to the documentation available from the compound suppliers, have a (Z) configuration.

In the Gal4-RORγ luciferase assay, two of the four follow-up compounds (i.e., **15.2** and **15.3**) were confirmed to function as inverse agonists of RORγ (IC_50_ values of 3.1 μM and 2.7 μM, respectively; Figure 3A and Table 1) at an activity level comparable to that of **15** (IC_50_ of 0.4 μM). The *I*_max(%)_ values of **15.2** and **15.3** were 58% and 53% in the Gal4-RORγ luciferase assay, respectively. Similar behavior and activities were measured in the full-length RORγ luciferase assay (Figure 3B and Table 1).

Consistent with the results obtained for **15** and betulinic acid, no cytotoxic effects were observed for any of the follow-up compounds in the resazurin assay at 5 μM (Figure S1A). Importantly, although they were not cytotoxic at 5 μM, the newly identified lupane-type triterpenoids exhibited cytotoxic effects at higher concentrations, which hindered our ability to test them further. None of the compounds investigated inhibited luciferase enzyme activity at 5 μM concentration (see the Experimental Section for details).

### Thermal Stability

To confirm the binding of betulinic acid and the newly discovered compounds to the human RORγ (hRORγ) LBD in a cell-free setting, nano differential scanning fluorimetry (nanoDSF) was employed.^66^ As shown in Figure 5, all tested ligands stabilize hRORγ LBD, with thermal shift (ΔTm) values ranging from 1.1 to 9.6 °C (Table 1). In comparison, the published RORγ inverse agonist T0901317 ^67^ stabilized the hRORγ LBD by 10.4 °C. The strongest stabilizations were observed for betulinic acid (ΔTm of 9.1 °C) and **15** (ΔTm of 9.6 °C). Despite the differences in the strengths of their inverse agonistic activities, the thermal shifts observed for these two compounds are comparable. Compound **15.1** also strongly stabilized hRORγ LBD (ΔTm of 5.9 °C), despite weak inverse agonistic activities in luciferase assays. In contrast, **15.2** and **15.3** exhibited lower thermal shifts (ΔTm of 2.2 and 2.8 °C, respectively), despite showing good activities in the Gal4-RORγ luciferase assay. For **15.4**, the thermal shift was just 1.1 °C, indicating low, if any, binding and stabilization. This result is in agreement with the results of the luciferase assay, which shows a lack of inverse agonistic activity of **15.4**.

**Figure 5:**
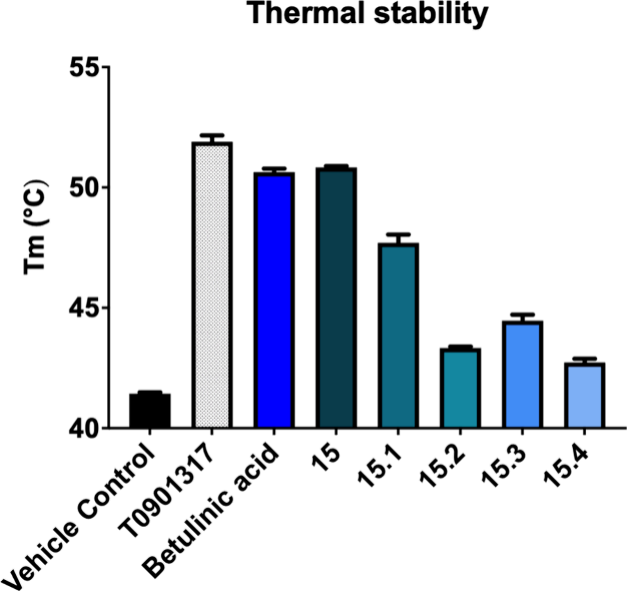
Thermal stabilization hRORγ LBD upon ligand binding. nanoDSF was used to assess the thermal stability of the purified apo hRORγ LBD and its change upon ligand binding.

Discrepancies between nanoDSF thermal shift data and luciferase assay results likely arise due to differences in assay readouts and biological context. NanoDSF evaluates direct ligand-induced stabilization of the purified hRORγ LBD in vitro, whereas the luciferase gene reporter assay reflects functional transcriptional activity within a cellular environment. Factors such as coregulator recruitment and the ligand’s ability to enter cells and the nucleus may contribute to the observed differences.^68^

### Binding Mode Prediction

Our docking experiments with Glide, using an X-ray crystal structure of the hRORγ-LBD in complex with ursonic acid (PDB structure 6J3N), indicate that betulinic acid, **15**, **15.2**, **15.3,** and **15.4** likely share the same binding mode, as shown in Figure 6A and B. The docking scores computed for the compounds ranged from −9.8 kcal/mol (for betulinic acid) to −13.4 kcal/mol (for **15.2**), suggesting favorable interactions between the compounds and the residues forming the ligand binding site (Table 1).

**Figure 6:**
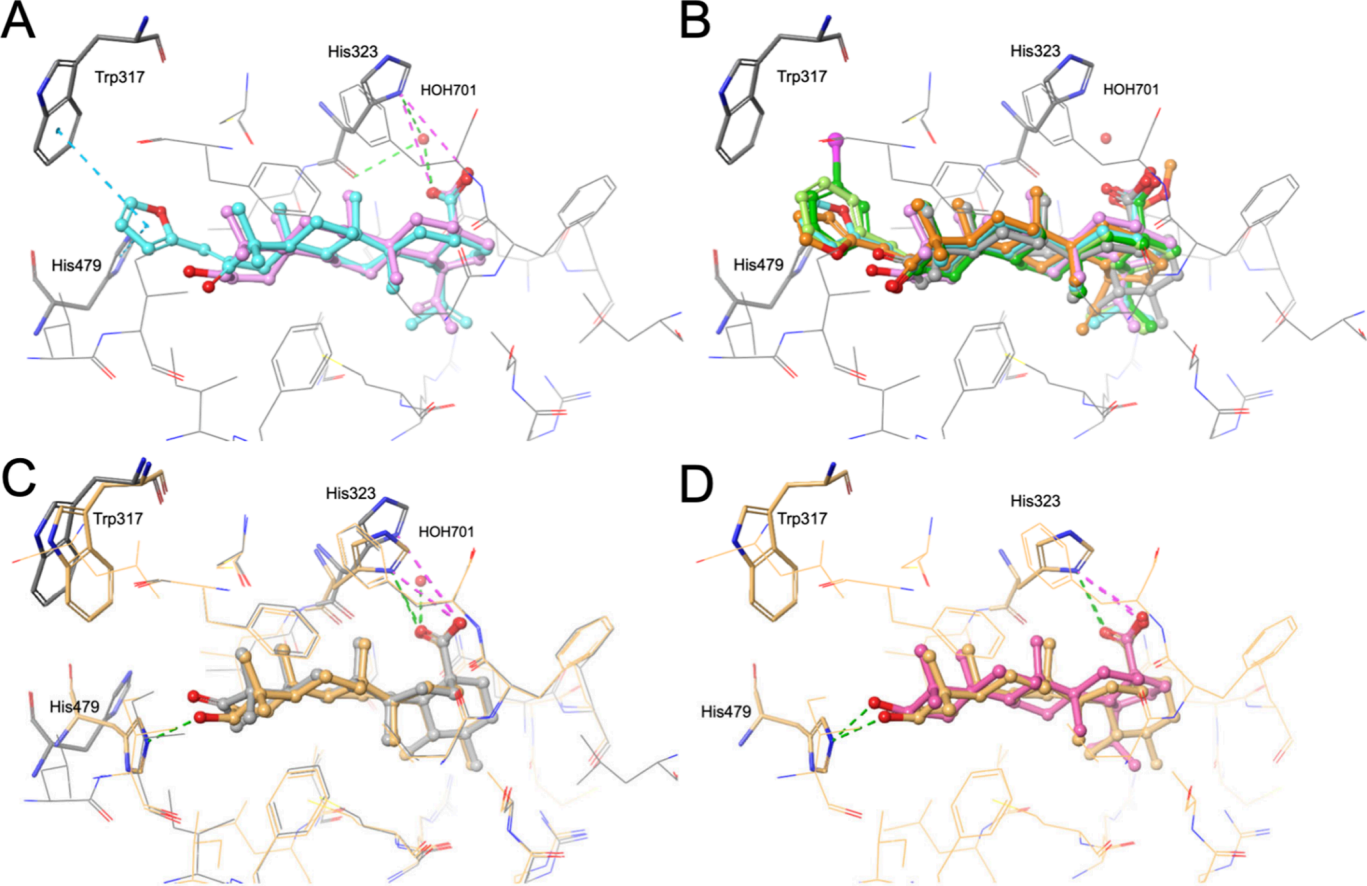
(A, B) Prediction of the binding modes of betulinic acid (depicted in A and B; plum carbon atoms), **15** (depicted in (A) and (B); cyan carbon atoms), and the follow-up compounds (depicted in (B); **15.2** with dark green carbon atoms, **15.3** with light green carbon atoms and **15.4** with orange carbon atoms) to the human RORγ-LBD (PDB structure 6J3N; cocrystallized ligand ursonic acid depicted in B with gray carbon atoms). (C) Comparison of binding modes of PDB structures 6J3N (protein structure and cocrystallized ligand ursonic acid depicted with gray carbon atoms) and 5X8S (protein structure and cocrystallized ligand ursolic acid depicted with light orange carbon atoms). (D) Alternative binding mode of betulinic acid (pink carbon atoms) to the human RORγ-LBD (result obtained by docking with PDB structure 5X8S; cocrystallized ligand ursolic acid depicted with light orange carbon atoms). In all panels, residues predicted to engage in important interactions with the ligands are marked and highlighted by a thick tube representation. Predicted hydrogen bonds, salt bridges, and pi-pi stacking are indicated by green, magenta, and cyan dashed lines, respectively. Interactions are not shown in panel B for better visualization.

According to the docking poses, the compounds are expected to form hydrogen bonds with His323 via water molecule 701. Some X-ray structures (e.g., 5X8S^69,70^ ) indicate conformational flexibility of the His323 side chain, which could also allow it to form a salt bridge with the ligand’s carboxylic acid moiety (Figure 6C). The absence of activity of **15.4** supports the essential role of the carboxylic acid moiety in ligand binding.

In addition to interactions with His323, **15**, **15.2**, **15.3,** and **15.4** are expected to form pi-pi stacking interactions with Trp317 and His479 with their furan or phenyl moieties. These interactions could break the His479-Tyr502-Phe506 agonist lock, which is related to RORγ-dependent transcriptional activation, by allowing it to adopt a suitable conformation for coactivator binding.^71^ Importantly, the described pi-pi interactions are not expected to be formed with betulinic acid because of the missing substituent on position 2.

Compound **15.1** features a distinct double bond configuration at position 2 of the triterpene scaffold (i.e., the E-configuration), whereas all other analogs possess the Z-configuration. Docking indicates that, unlike the (Z) isomer, the (E) isomer likely does not fit into the ligand binding pocket.

### Site-Directed Mutagenesis

The predicted binding modes suggest significant interactions between the compounds and the residues His323 and His479. To further investigate the predicted binding mode, we introduced two mutations into the RORγ-LBD: His323Ala (H323A) and His479Leu (H479L). Both mutants were confirmed to be transcriptionally active, as indicated by their relative luminescence unit (RLU) values in the luciferase assay (Figure S2). Consistent with the docking predictions, all compounds lost activity when tested on Gal4-RORγ H323A, except **15.4**, which was already inactive on the wild-type (WT) nuclear receptor (Figure 7A).

**Figure 7:**
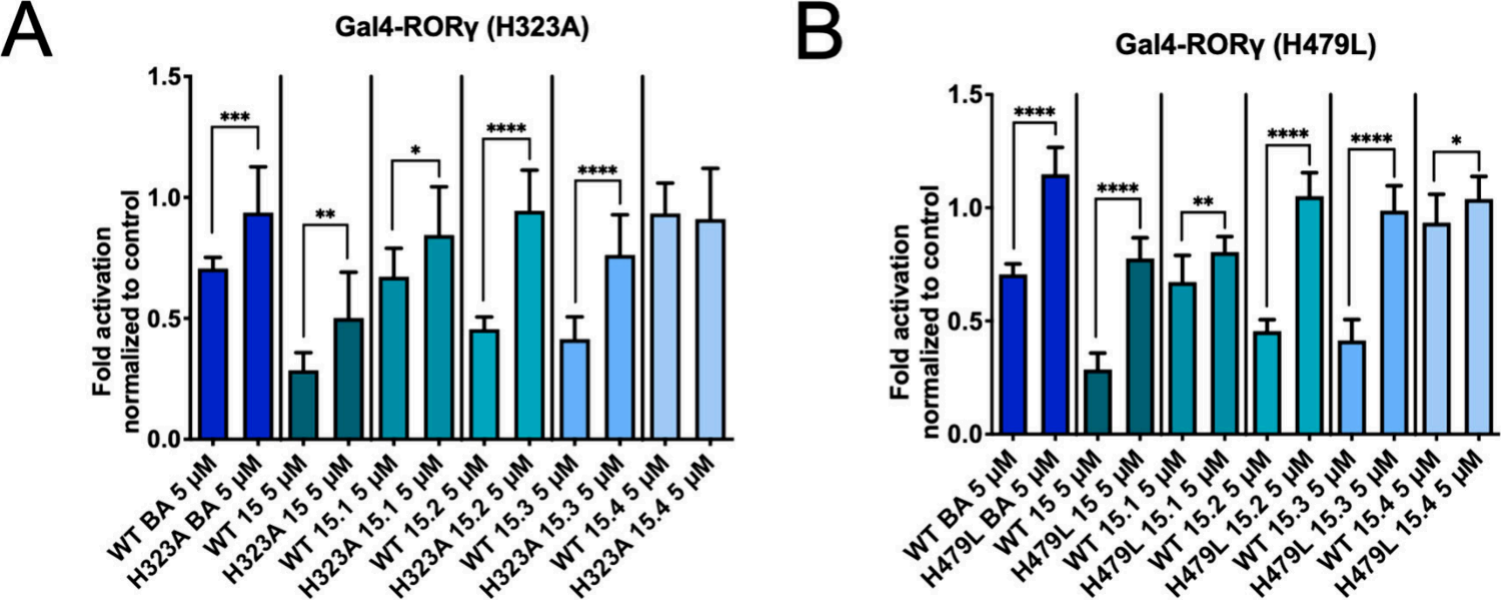
Experimental evaluation of betulinic acid and **15** to **15.4** on the Gal4-RORγ H323A (A) and H479L (B) mutants. Compounds were tested at 5 μM concentration in cell-based Gal4-RORγ luciferase assays, where the mutations were introduced into the RORγ-LBD through site-directed mutagenesis. The luminescence signals derived from the luciferase reporter were normalized to eGFP fluorescence and expressed as fold activation normalized to the vehicle control. Bar charts represent transactivation activities as mean ± SD of at least three biological replicates (*n* ≥ 3) measured in technical quadruplicates. An unpaired two-tailed *t* test was used for statistical analysis. ****p ≤ 0.0001, ***p ≤ 0.001, **p ≤ 0.01, *p ≤ 0.05, no indication *p* > 0.05 (activity of a compound on WT vs mutant receptor).

Testing the compounds on the H479L mutant revealed that most (e.g., **15**, **15.2**, and **15.3**) experienced a loss of activity (Figure 7B), as inferred from the predicted complexes. Compound **15.4** remained inactive.

Surprisingly, betulinic acid showed a notable decrease in activity when tested on the H479L mutant. This may be due to the adaptability of the hydroxyl group at position 3 in forming a hydrogen bond interaction with His479 (the predicted binding pose with PDB structure 5X8S is shown in Figure 6D). Lastly, we also assessed the activity of our positive control, SR2211, on both mutants (Figure S3). In each case, a clear loss of activity was observed. This can be rationalized by the crystal structure of the RORγ LBD complexed with SR2211 (PDB 6NWT),^72^ which shows that SR2211 forms a hydrogen bond with His479 and is positioned close to His323, suggesting potential stabilizing interactions.

### Biological Characterization with RT-qPCR

To further explore the downstream biological impact of betulinic acid and **15** to **15.4** as RORγ inverse agonists, we performed RT-qPCR utilizing EL-4 cells transfected with mRORγt (EL-4-mRORγt cells). We chose *Il17a* and *Il17f* (summarized as *Il17a/f* in this section) as our RORγ target genes of interest and the inverse agonist SR2211 as the RORγ-selective positive control.

As expected from a selective inverse agonist of RORγ, and in agreement with the literature,^26,28^ the positive control SR2211 led to a significant and reproducible down-regulation of *Il17a/f* expression in all experiments, proving the functionality of our assay (Figures 8 and S4). In accordance with luciferase data, **15.1** and **15.4** were either inactive (**15.1**; Figure S4A) or slightly but significantly up-regulated *Il17a/f* levels (**15.4**; Figure S4B). Compounds **15.2** and **15.3** led to a significant and concentration-dependent reduction of *Il17a/f* expression (Figure 8A and B, respectively), as did betulinic acid at 5 μM (Figure S4C). These results are in line with those gathered by luciferase assays. Unexpectedly, however, our most active compound in the luciferase assays, **15**, failed to affect *Il17a* expression levels but led to a slight yet concentration-dependent decrease in *Il17f* expression (Figure 8C). To resolve the ambiguity in this specific case, we checked the ability of **15** to regulate a third RORγ target gene, interleukin 23 receptor (*Il23r*).^28,73^ In line with our observations concerning *Il17f*, **15** led to a concentration-dependent down-regulation of *Il23r* (Figure 8D). Recent reports of context-specific activities of RORγ inhibitors by Zou and colleagues might explain the lack of activity of **15** on Il17a expression.^26^ They found that inverse agonists of RORγ could show inhibitory, agonistic, or no biological effect, depending on (i) the cells used, (ii) the target program explored (e.g., cholesterol biosynthesis vs cytokine expression), or (iii) the target templates employed (e.g., plasmid-based reporter vs natural chromatin). They linked these findings to differential alterations of local chromatin structure at the target loci of RORγ.^26^ Since we were able to exclude luciferase enzyme inhibition and cytotoxic effects of our compounds in HEK293 and EL-4-mRORγt cells (Figure S1A and B, respectively), it is conceivable that similar effects could be in place in our study as well. This may explain the strong inhibitory activity of **15** observed in the luciferase assays, despite its lack of downstream effect on *Il17a* gene expression.

**Figure 8:**
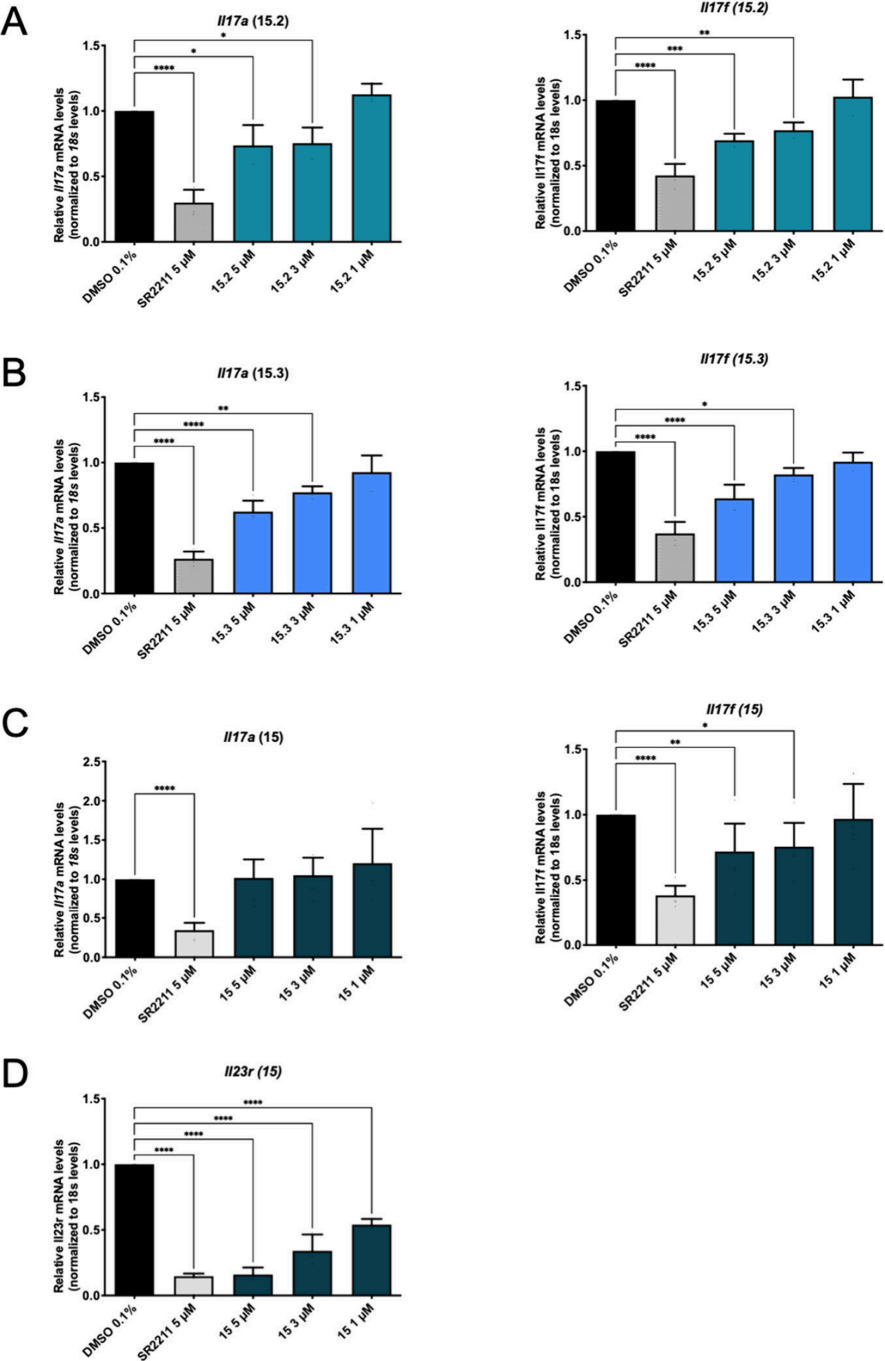
RT-qPCR analysis of **15.2**, **15.3**, and **15** in EL-4-mRORγt cells. (A–D) Analysis of *Il17a/f* and *Il23r* (the latter only in case of **15**) gene expression levels in EL-4-mRORγt cells pretreated with vehicle control, the positive control SR2211 (5 μM) or the compounds of interest at the indicated concentrations for 20–24 h. Treatment was followed by a 4.5-h stimulation with PMA and ionomycin, after which total RNA was isolated, reversely transcribed into cDNA, and subjected to RT-qPCR. Data are presented as mean ± SD from at least three biological replicates (*n* ≥ 3) performed in technical duplicates or triplicates. One-way ANOVA, followed by Dunnett’s post hoc test, was used for statistical analysis. ****p ≤ 0.0001, ***p ≤ 0.001, **p ≤ 0.01, *p ≤ 0.05, no indication *p* > 0.05 as compared to vehicle control.

IL-23R is essential for the terminal differentiation of Th17 cells, and a lack of IL-23R signaling leaves Th17 cells unable to mediate encephalitogenicity in experimental autoimmune encephalomyelitis.^74^ Moreover, IL-23R signaling plays a crucial role in granulocyte-macrophage colony-stimulating factor expression (together with RORγ)^75^ and IFN-γ induction in Th17 cells,^76,77^ underpinning its major pathogenic role (reviewed in ref 78). Furthermore, it was shown that dual inhibition of IL-17A and IL-17F with bimekizumab was more effective compared to inhibition of IL-17A alone (with secukinumab) in patients with moderate-to-severe psoriasis,^79^ suggesting that a down-regulation of more than one target gene at the same time (i.e., *Il17a/f* in case of **15.2** and **15.3** or *Il17f* and *Il23r* in case of **15**) could prove beneficial. In summary, we could confirm changes in RORγ target gene expression for the active RORγ inverse agonists identified in the luciferase assays, except for **15** on *Il17a*.

## Conclusions

By docking 113,687 compounds of MolPort’s Database of Purchasable Natural Compounds to the RORγ-LBD, we identified **15**, a lupane-type triterpenoid structurally related to betulinic acid, as a new RORγ inverse agonist. Compound **15** exhibited potent inverse agonist activity, with IC_50_ values of 0.4 μM and 0.9 μM as determined by Gal4-RORγ and full-length RORγ luciferase assays, respectively. The *I*_max(%)_ values at 5 μM were 76% and 69%, respectively. In contrast, betulinic acid showed only moderate activity in the same assays (*I*_max(%)_ values of 47% and 38% at 5 μM concentration, respectively). Four follow-up compounds structurally related to **15** were purchased and tested. Two of these were confirmed to act as RORγ inverse agonists, albeit with a weaker potency and efficacy. Furthermore, employing RT-qPCR, we confirmed that the three newly discovered compounds (i.e., **15**, **15.2,** and **15.3**) decreased the expression of RORγ target genes. None of the investigated compounds showed cytotoxicity at a 5 μM concentration in resazurin conversion assays, and none of the compounds inhibited luciferase activity. Nano differential scanning fluorimetry confirmed the binding and stabilization capacity of the tested compounds to the RORγ LBD. The binding modes predicted by docking suggest that the carboxylic acid group and an additional aromatic ring substitution in the correct configuration present in **15** are key contributors to its high potency. By employing site-directed mutagenesis, we confirmed the importance of His323 and His479 within the RORγ-LBD for the binding of betulinic acid and the other lupane-type triterpenoids.

While these findings are encouraging, they also highlight the critical next steps needed to advance this research. First, the established structure–activity relationship should guide the rational design of new analogs with enhanced potency and reduced cytotoxicity, with a particular focus on achieving specificity for RORγ. Following this, comprehensive selectivity profiling is essential to confirm target specificity. Finally, the optimized and selective compounds should be assessed in functional assays, such as the inhibition of Th17 cell differentiation, to validate their efficacy in a biologically relevant context.

In summary, the compounds presented in this study broaden the scope of triterpenoids as modulators of RORγ. By exploring various substitutions at position 2 of the lupane scaffold, we have identified a new and effective structural motif for RORγ inverse agonism. The resulting structure–activity relationship offers guidance for the rational design of more potent and selective derivatives. This work provides a strong foundation for the development of triterpenoid-based therapies targeting Th17-driven diseases such as psoriasis and rheumatoid arthritis.

## Experimental Section

### General Experimental Procedures

Human RORγ protein structures were retrieved from the Protein Data Bank (PDB),^80,81^ filtered based on quality criteria, and prepared with Schrödinger’s Protein Preparation Wizard. Molecules from the MolPort Natural Product Library were prepared using LigPrep and docked into selected RORγ crystal structures using Glide SP mode.

Reagents and solvents were obtained from commercial suppliers. Compounds were dissolved in DMSO, stored at −70 °C, and diluted in methanol for analytical verification. Identity and purity were confirmed using an ultra high pressure liquid chromatography (UHPLC) coupled to a diode array detector (DAD), corona aerosol detector (CAD), and mass spectrometer (MS) system (UHPLC-DAD-CAD-MS system; Thermo Fisher Scientific, San Jose, CA, USA).

HEK293 and EL-4 cells were cultured in DMEM with 10% FBS and antibiotics; HEK293 cells were transfected with reporter constructs and analyzed using a Tecan Spark spectrophotometer. Site-directed mutagenesis was performed with the QuikChange Lightning Kit, plasmids were prepared using the PureLink HiPure Midiprep Kit, and verified by Sanger sequencing (Microsynth). mRORγt was PCR-amplified using the Herculase II Fusion DNA Polymerase kit with SnapGene-designed primers, gel-purified, digested with NheI-HF/XhoI, ligated with T4 DNA Ligase, and cloned into pIRES2-eGFP; plasmids were verified by Sanger sequencing. EL-4 cells were transfected with AseI-linearized vector using Lipofectamine LTX, sorted twice for GFP^+^ cells using a CytoFLEX SRT, and analyzed with a MACSQuant Analyzer 10 and FlowJo. For viability, cells were treated with compounds and resazurin in phenol red-free DMEM. The His-tagged hRORγ LBD was expressed in *E. coli* BL21 DE3, purified using Ni Hitrap FF and Superdex 200 columns, concentrated with Amicon Ultra filters, and analyzed for thermal stability using Prometheus NT.48 and PR.Stability Analysis.

### Preparation of Protein Structures for Docking

The PDB was queried for all structures of human RORγ using UniProt accession P51449. This search resulted in a list of 151 PDB entries (all of these structures are derived from X-ray experiments), of which any structure matching any of the following criteria was removed:

- Resolution > 2 Å,
- electron density map not available, and
- no inverse agonist bound to the orthosteric site (according to information retrieved from the primary literature).

The remaining 16 protein structures were prepared with the Protein Preparation Wizard (part of the Schrödinger Platform).^82,83^ As part of this procedure, missing side chains were added, solvent molecules and crystallization aids were removed, the H-bond network was optimized, and the protein structure was minimized (all settings were kept at their default values). The binding sites were aligned, and four representative structures were selected for docking with Glide considering the conformation of the binding sites and ligand diversity. In preparation for docking, receptor grids were generated for each chain A of the four protein structures with the Receptor Grid Generation wizard (part of the Schrödinger Platform). The location and size of the individual grids were defined based on the cocrystallized ligand using default settings.

### Preparation of Small Molecules for Docking

The molecular structures included in the MolPort Natural Product Library were prepared for docking with LigPrep (part of the Schrödinger Platform).^84^ More specifically, (i) molecules with more than 90 heavy atoms were removed, (ii) Epik^85,86^ (also part of the Schrödinger Platform) was employed to assign ionization states at pH 7 ± 0, (iii) salts were removed, (iv) unspecified configurations of chiral atoms were enumerated (up to a maximum of 32 stereoisomers per compound), and (v) geometries were optimized with the OPLS4 force field^87^ applying default settings.

### Docking

Virtual screening was performed with Glide (part of the Schrödinger Platform for drug discovery, version 2021–1) in Standard Precision mode (Glide SP). All compounds in the prepared MolPort Natural Product Library were docked against the four binding pockets. Up to four docking poses were stored and inspected for each binding pocket and compound.

Binding mode analysis followed the same protocol as the virtual screening approach. However, up to 100 docking poses were generated instead of four, and a new version of the Schrödinger Platform (i.e., version 2023–4)^88^ was used.

### Cell Lines, Plasmids and Chemicals

Human embryonic kidney 293 (HEK293) cells (CRL-1573) were acquired from the American Type Culture Collection (ATCC; Manassas, VA, USA). EL-4 cells (murine T lymphoblasts; 85023105) were obtained from the European Collection of Authenticated Cell Cultures (ECACC). DMEM with 4.5 g/L glucose and without phenol red (12–917F), l-glutamine (BE17–605E), and the penicillin-streptomycin mixture (DE17–602E) were purchased from Lonza (Basel, Switzerland). DMEM with 4.5 g/L glucose and phenol red (D6546), SR2211 (SML1170), betulinic acid (B8936), digitonin (D141), coenzyme A (CoA) trilithium salt (C3019), DL-dithiothreitol (DTT; 43815), and resazurin sodium salt (199303) were acquired from Sigma-Aldrich (St. Louis, MO, USA). Fetal bovine serum (FBS; S1810; batch number S00CN) was obtained from Biowest (Nuaillé, France). Trypsin (27250–018), Lipofectamine LTX Reagent with PLUS Reagent (12343593), the High-Capacity cDNA Reverse Transcription Kit (4368814), the PureLink HiPure Plasmid Midiprep Kit (K210005), SYBR Safe DNA Gel Stain (S33102), the GeneRuler 1 kb Plus DNA Ladder, ready-to-use (SM1333), and Zeocin (J67140.8EQ) were purchased from Thermo Fisher Scientific (Waltham, MA, USA). Dimethyl sulfoxide (DMSO; 4720.2), ethylenediaminetetraacetic acid (EDTA; 8043.2), adenosine-5′-triphosphate (ATP) disodium salt (HN35.2), LB Broth (X964.2), and ampicillin sodium salt (HP62.1) were acquired from Carl Roth (Karlsruhe, Germany). Reporter Lysis 5X Buffer (E4030) was obtained from Promega (Fitchburg, WI, USA). D-luciferin sodium salt (BC218) was purchased from SynChem (Elk Grove Village, IL, USA). eGFP-N1 (6085–1) and pIRES2-eGFP (6029–1) were acquired from Clontech (Kusatsu, Japan). All other plasmids (Gal4-hRORγ, pTK-MH100 × 4-LUC, full-length human RORγ, RORE-LUC, and full-length murine RORγ) were kindly gifted to us by providers listed in the Supporting Information (Table S2). All primers used in this work were synthesized at Microsynth (Balgach, Switzerland) and are listed in Table S3. The QuikChange Lightning Site-Directed Mutagenesis Kit (210519), XL1-Blue Competent Cells (200249), and the Herculase II Fusion DNA Polymerase (600677) were obtained from Agilent (Santa Clara, CA, USA). Agarose (AGA500-BCAT) was purchased from BioCat (Heidelberg, Germany). Cell Activation Cocktail (without Brefeldin A; 423301) was acquired from BioLegend (San Diego, CA, USA). The innuPREP RNA Mini Kit 2.0 (845-KS-2040010) was obtained from IST Innuscreen GmbH (Berlin, Germany). The Luna Universal qPCR Master Mix (M3003), the Monarch DNA Gel Extraction Kit (T1020S), NheI-HF (R3131S), XhoI (R0146S), AseI (R0526S), and the T4 DNA Ligase (M0202S) were purchased from New England Biolabs (NEB; Ipswich, MA, USA). Compounds **1** (Y042–6511) and **2** (C073–4674) were acquired from ChemDiv (San Diego, CA, USA). Compounds **3** (STL522502), **4** (STK996882), **5** (STK099349), **6** (STL476697), **7** (STL532829), **8** (STK642752), **9** (STL536931), **10** (STL517425), **11** (STL496064), **12** (STL529732), **13** (STL536614), **14** (STL465232), **15** (STL526289), **15.1** (STL526053), **15.2** (STL526067), **15.3** (STL526337), **15.4** (STL526398), **16** (STL034255), **17** (STL535026), **18** (STL527184), **19** (STL522764), **20** (STL535149), **21** (STK860045), **22** (STL565695), **23** (STK612635) were obtained from Vitas-M Laboratory (Hong Kong, People’s Republic of China) via MolPort. Methanol (MeOH; 83638.320) was purchased from VWR (Radnor, PA, USA). T0901317 (2373) was purchased from Tocris Bioscience (Bristol, UK).

### Compound Stocks, Dilutions, and Identity/Purity Checks

All compounds were dissolved in 100% DMSO under sterile conditions (HERAsafe KS18, Thermo Fisher Scientific, Waltham, MA, USA). Compound stocks and dilutions were stored at −70 °C. For identity and purity checks, compound stocks (dissolved in DMSO) were diluted 1:100 with MeOH before analysis.

Identity and purity checks were performed using a UHPLC-DAD-CAD-MS system -UHPLC (ultrahigh pressure liquid chromatography) coupled to three detectors: 1) UV-DAD (diode array detector), 2) CAD (corona aerosol detector), and 3) MS (mass spectrometer). The Ultimate 3000 UHPLC-DAD-CAD system (Thermo Fisher Scientific, San Jose, CA, USA) was equipped with a reversed-phase ACQUITY UPLC CSH C18, 130Å, 1.7 μm, 2.1 mm × 100.0 mm column (Waters, Framingham, MA, USA). Mobile phases A (H2O/FA, 100:0.01) and B (ACN) were degassed before usage. A 10 min binary gradient with flow rate set to 350 μL/min was applied as follows: 0–1 min, 80% mobile phase B; 2–6 min, 80–98% mobile phase B; 6–8 min, 98% mobile phase B; 8–10 min re-equilibration with 80% mobile phase B). Ten microliters of each compound were injected, followed by a blank (Mobile phase only) injection to ensure proper column washing and equilibration. DAD and CAD detection provided the chromatograms used to assess the purity of the compounds. Mass spectrometric detection, to confirm the identity of the compounds, was performed with an LTQ-XL linear ion trap mass spectrometer (Thermo Fisher Scientific, San Jose, CA, USA) using the HESI source (350 °C capillary temperature, 54/12/3 arb. units for the sheath, aux, and sweep gases respectively and 3.5 Kv spray voltage) to achieve negative/positive ion mode ionization. MS scans were performed with an *m*/*z* range from 100 to 2000 Da.

### Cell Culture

All steps involving cells were performed under sterile conditions. HEK293 and EL-4 cells were cultured in DMEM with 4.5 g/L glucose and phenol red, supplemented with 10% FBS, 2 mM l-glutamine, 100 U/mL penicillin, and 100 μg/mL streptomycin (“complete DMEM”). Incubations took place at 37 °C and 5% CO_2_. Cells were passaged every 2–3 days and used up to in-house passage number 30. Cell concentration and viability were determined using an automated cell counter (Vi-CELL XR Cell Viability Analyzer, Beckmann, Krefeld, Germany). When indicated, DMEM with 4.5 g/L glucose and phenol red was supplemented with 5% charcoal-stripped FBS (“stripped DMEM”) instead of 10% FBS. All other components stayed the same. When indicated, stripped DMEM was prepared using DMEM with 4.5 g/L glucose without phenol red (“stripped DMEM without phenol red”).

### Luciferase Reporter Gene Assay

For luciferase assays, 8 × 10^6^ HEK293 cells were seeded on 150 mm cell culture dishes and incubated for 5 h. Afterward, cells were transfected with the calcium phosphate coprecipitation method^89^ using 5 μg of the nuclear receptor (Gal4-hRORγ or full-length human RORγ), 5 μg of a luciferase reporter (pTK-MH100 × 4-LUC for Gal4-hRORγ experiments or RORE-LUC for full-length human RORγ experiments) and 3 μg eGFP-N1 (for assessing transfection efficiency) plasmid DNA and incubated overnight. In some cases, cells were only transfected with pTK-MH100 × 4-LUC (5 μg) and eGFP-N1 (3 μg) to check whether a compound inhibited luciferase directly. The medium was changed the following day, and cells were further incubated for another four to 5 h before trypsin/EDTA was added to detach them from the dishes. Complete DMEM was added to stop trypsinization, and cell suspensions were centrifuged at 410 × g for 4 min. Cell pellets were resuspended in stripped DMEM and seeded at a density of 5 × 10^4^ cells per well in a 96-well plate before treatment with the vehicle control (0.1% DMSO), the positive control (SR2211) or the compounds of interest at the indicated concentrations for 18 h. Subsequently, cells were checked microscopically for signs of toxicity and compound precipitation, frozen at −70 °C for 1 h, and lysed using the “Reporter Lysis 5X Buffer”, which was diluted to 1X with H_2_O and supplemented with 450 μM CoA and 5 mM DTT. Fluorescence and luminescence values (the latter after applying 50 μL ATP and D-luciferin via injectors A and B, respectively) were measured using a spectrophotometer (Tecan Spark, Tecan Group, Männedorf, Switzerland). RLUs were normalized to relative fluorescence units (RFU) to account for differences in cell number and transfection efficiency and then normalized to the vehicle control. Results are expressed as fold activation relative to the vehicle control.

### Site-Directed Mutagenesis

Gal4-hRORγ H323A and H479L mutants were generated using the QuikChange Lightning Site-Directed Mutagenesis Kit according to the manufacturer’s instructions. Briefly, 10 ng template DNA (Gal4-hRORγ) was PCR-amplified with the respective mutagenic forward and reverse primers listed in Table S3, which were designed using the QuikChange Primer Design tool (https://www.agilent.com/store/primerDesignProgram.jsp). PCR settings are listed in Table S4. Afterward, the methylated template DNA was DpnI-digested at 37 °C for 5 min. Two μL of the PCR product were then transformed into XL1-Blue competent *E. coli* according to a protocol provided by NEB.^90^ After overnight incubation at 37 °C, single colonies were picked, transferred into 5 mL lysogeny broth (LB) medium containing 50 μg/mL zeocin, and shaken for 5 h at 37 °C (preculture). The preculture was then added to 400 mL LB medium containing 50 μg/mL zeocin and shaken at 37 °C overnight (main culture). After centrifuging the main culture at 5691*g* for 10 min, the supernatant was decanted, and plasmid preparation was performed using the PureLink HiPure Plasmid Midiprep Kit according to the manufacturer’s instructions. DNA yields and purities were checked using a NanoDrop 2000c (Thermo Fisher Scientific, Waltham, MA, USA). The presence of the correct mutations was confirmed by Sanger sequencing at Microsynth using their “GAL4-BD” standard primer or the self-designed sequencing primer “RORg-Gal4 LBD sequencing fwd 1” (both listed in Table S3) for the H323A and H479L mutations, respectively.

### Construction of an mRORγt-pIRES2-eGFP Vector

For construction of the mRORγt-pIRES2-eGFP vector, mRORγt was first PCR-amplified out of the full-length murine RORγt pcDNA3.1 vector using the Herculase II Fusion DNA Polymerase kit according to the manufacturer’s instructions. NheI and XhoI restriction sites were added to the fragment’s 5′ and 3′ ends, respectively. For this, the primers “mRORgt pIRES2-eGFP NheI fwd” and “mRORgt pIRES 2-eGFP XhoI rev” (Table S3) were designed using SnapGene Viewer (Dotmatics, Boston, MA, USA). PCR conditions are listed in Table S5. The PCR product was separated on a 1% agarose gel containing 0.5X SYBR Safe, cut out under UV light, and extracted using the Monarch DNA Gel Extraction Kit according to the manufacturer’s instructions. After double digestion of the fragment and pIRES2-eGFP vector with NheI-HF and XhoI according to a protocol provided by NEB,^91^ the fragment and vector were again separated on a 1% agarose gel containing 0.5X SYBR Safe and extracted using the Monarch DNA Gel Extraction kit. For ligation estimation, 0.2, 0.5, and 1 μL of cut fragment and vector were separated on a 1% agarose gel containing 0.5X SYBR Safe. The bands were quantified using ImageJ (National Institutes of Health, Bethesda, MD, USA) and compared with the intensity of the 1500 bp band of the calibrator (GeneRuler 1 kb Plus DNA Ladder, ready-to-use). Ligation was done using the T4 DNA Ligase according to a protocol provided by NEB,^92^ using a 7x molar excess of the fragment relative to the vector. In general, transformation and plasmid preparation of the ligation product were performed as described in the “Site-directed mutagenesis” section, with the exceptions that 5 μL of product was used instead of 2 μL for initial transformation and that ampicillin (50 μg/mL) was used as the selection antibiotic instead of zeocin. Correct insertion of mRORγt inside the vector was confirmed by Sanger Sequencing at Microsynth using their “IRES-for” standard primer (Table S3).

### Transfection and Fluorescence-Activated Cell Sorting of EL-4 Cells

Transfection of EL-4 cells with the AseI-linearized^93^ mRORγt-pIRES2-eGFP vector was done using Lipofectamine LTX according to the manufacturer’s instructions. GFP^+^ cells were sorted using a CytoFLEX SRT (Beckman Coulter, Brea, CA, USA) and cultured in complete DMEM until they reached confluency. Afterward, these cells were sorted a second time for GFP^+^ cells. After growing to confluency again, the percentage of GFP^+^ cells was checked using a MACSQuant Analyzer 10 flow cytometer (Miltenyi Biotec, Bergisch Gladbach, Germany) and confirmed to be >95% (Figure S5). Flow cytometric data was analyzed using FlowJo 10.8.1. (BD, Franklin Lakes, NJ, USA). Aliquots of these cells (referred to as “EL-4-mRORγt” cells) were frozen and kept in liquid nitrogen until use.

### Resazurin Conversion Assay

To check the metabolic activity of cells correlating with cell viability, cells were treated with the vehicle control (0.1% DMSO), digitonin as the positive control for cytotoxicity at 20 μg/mL (HEK293 cells) or 50 μg/mL (EL-4-mRORγt cells), or the compounds of interest at 5 μM for 18 h (HEK293 cells) or 20–24 h (EL-4-mRORγt cells). The next day, the medium was carefully removed and replaced with stripped DMEM without phenol red, containing 10 μg/mL resazurin sodium salt. Cells were incubated for 5 h before RFU values were measured at λ_em_ = 590 nm using a spectrophotometer. Cell viability was calculated as follows:

$$\mathrm{Cell viability}\;(\%)=100\times \frac{\mathrm{RFU value after compound treatment}}{\mathrm{RFU value after vehicle control treatment}}$$

Cytotoxicity was defined as cell viability under 70% according to the ISO 10993–5 guidelines.^94^ Cell viability <90% is explicitly stated in the respective figure.

### Thermal Stability (Nanoscale Differential Scanning Fluorimetry, nanoDSF)

The sequences encoding the His-hRORγ (264–518) LBD were inserted into the pET15b expression plasmid. The protein was produced in *Escherichia coli* BL21 DE3 by induction with 1 mM IPTG (Euromedex) at an OD600 of ∼ 0.8 and incubation at 22 °C for 2 h 30 min. Soluble proteins were purified on Ni Hitrap FFcrude column (Cytiva), followed by His tag removal by thrombin (MP Biomedicals) cleavage and by size exclusion chromatography on HiLoad Superdex200 column (Cytiva) equilibrated in 20 mM Tris-HCl pH 7.5, 150 mM NaCl, 1 mM TCEP (Hampton Research). Purity and homogeneity of the hRORγ LBD were assessed by SDS-PAGE. The purified protein was concentrated to 0.5–1.0 mg/mL with an Amicon Ultra 10 kDa MWCO. The aliquots of purified LBD were incubated with 4 equiv ligands or vehicle for thermal stability experiments.

Fluorescence-based thermal experiments were performed using a Prometheus NT.48 (NanoTemper Technologies, Germany) with grade standard capillaries containing 10 μL of RORγLBD with different ligands. The temperature was increased at a rate of 1 °C/min from 20 to 95 °C, and the fluorescence at emission wavelengths of 330 and 350 nm was measured for 3 technical replicates. NanoTemper PR. Stability Analysis v1.0.2 was used to fit the data and determine the melting temperatures Tm. The data presented is the average of 2 biological replicates (Table S6).

### Real-Time Quantitative Polymerase Chain Reaction (RT-qPCR)

For RT-qPCR assays, 5 × 105 EL-4-mRORγt cells were seeded in 500 μL complete DMEM per well of a 24-well plate and treated with the vehicle control, the positive control, or the compounds of interest. After incubation for 20–24 h, cells were stimulated with Cell Activation Cocktail (PMA: 40.5 μM, ionomycin: 669.3 μM; 500X) for 4.5 h. Afterward, total RNA was isolated using the innuPREP RNA Mini Kit 2.0 according to the manufacturer’s instructions. According to the manufacturer’s instructions, 1 μg RNA was reverse-transcribed into cDNA using the High-Capacity cDNA Reverse Transcription Kit. RT-qPCR was performed using the Luna Universal qPCR Master Mix on a LightCycler 480 (Roche Diagnostics, Rotkreuz, Switzerland) according to the manufacturer’s instructions. Measurement settings and used primers are listed in Tables S2 and S7, respectively. Primers were designed using Primer-BLAST (https://www.ncbi.nlm.nih.gov/tools/primer-blast/), and their efficiency testing revealed efficiencies of >95% for all of them. Livak’s 2−ΔΔCt method^95^ was employed to determine the expression levels of RORγ target genes. This involved normalizing the target gene expression to the expression levels of the control gene 18s,^96^ followed by further normalization to the vehicle control.

### Statistical Analysis of Bioactivity Measurements and Curve Fitting

All bioactivity measurements were independently performed at least three times with three (RT-qPCR) to four (luciferase and resazurin conversion assay) technical replicates per condition unless stated otherwise. Graphs were prepared, and statistical analyses were performed using GraphPad Prism software version 10 (Dotmatics, Boston, MA, USA). All measured bioactivity data are represented as mean ± standard deviation (SD) unless stated otherwise. To check for statistically significant differences between the measured bioactivities of compounds and the vehicle control DMSO, a one-way analysis of variance (ANOVA) with Dunnett’s posthoc test was performed. In instances where all experimental groups were compared pairwise, Tukey’s posthoc test was employed instead. To determine statistically significant differences between the measured activities of compounds on a WT relative to a mutant RORγ, an unpaired two-tailed *t* test was used. In both cases, statistical significance (i.e., the activity of a compound vs DMSO or activity of the same compound on a WT vs a mutant RORγ) is indicated as follows: ****p ≤ 0.0001, ***p ≤ 0.001, **p ≤ 0.01, *p ≤ 0.05, ns (or no indication) *p* > 0.05.

Concentration–response data were analyzed in GraphPad Prism version 10 by three-parameter logistic nonlinear regression, with the Hill slope constrained to a theoretical value of −1.0. An IC_50_ value was determined only for compounds yielding a statistically reliable fit. A fit was considered reliable if it met the following quantitative criteria: R^2^ > 0.5 and a 95% confidence interval of the IC_50_ spanning less than a 10-fold range. For compounds that failed to meet these statistical criteria, or for those that showed no clear concentration–response trend upon visual inspection, an IC_50_ was not determined. In these cases, only the maximal inhibition achieved at the highest tested concentration (*I*_max(%)_ at 5 μM) is reported, calculated as follows:

$$I\max\;\left(\%\right)=\left(1-\frac{\mathrm{Bottom}}{\mathrm{Top}}\right)\times 100$$

­(“top” = baseline activity measured at the lowest concentration, “bottom” = residual activity observed at the highest concentration, corresponding to the maximal inhibitory effect).

## Supplementary Material



## Notes

Results of resazurin conversion assays in HEK293 and EL-4 mRORγt cells, transactivation activity of Gal4-RORγ H323A and H479L mutants, impact of SR2211 on the transactivation activity of the two mutants, RT-qPCR analysis of **15.1**, **15.4**, and betulinic acid in EL-4-mRORγt cells, and flow cytometric analysis of EL-4-mRORγt cells, complete list of compounds selected from virtual screening for experimental evaluation, overview of plasmids and sources, list of primers used in this work, PCR settings for site-directed mutagenesis, PCR settings for amplification of murine RORγt and addition of NheI and XhoI restriction sites, overview of nanoscale differential scanning fluorimetry replicates, and measurement settings for RT-qPCR on the LightCycler 480 (PDF)
