# Supplementary material for: Identification of New Lupane-Type Triterpenoids as Inverse Agonists of RAR-Related Orphan Receptor Gamma (RORγ)
Source: J Nat Prod. 2025 Jul 28;88(8):1887–900. doi: 10.1021/acs.jnatprod.5c00416 (PMC12379159; doi:10.1021/acs.jnatprod.5c00416)
Supplement: Supplementary file 1 [file np5c00416_si_001.pdf]

Supporting Information

Identification of New Lupane-type Triterpenoids as Inverse Agonists of RAR-related Orphan Receptor Gamma (RORγ)

Patrik F. Schwarz[1,2], Alexander F. Perhal[1], Famke Guder[3], Jorge Enrique Hernández González[4,5], Kerrin Janssen[4,6], Ece Sağıroğlu[4], Ammar Tahir[1], Johannes Kirchmair[4], Natacha Rochel[3], Verena M. Dirsch[1], and Ya Chen[4*]

[1] Department of Pharmaceutical Sciences, Division of Pharmacognosy, Faculty of Life Sciences, University of Vienna, Josef-Holaubek-Platz 2, 1090 Vienna, Austria

[2] Vienna Doctoral School of Pharmaceutical, Nutritional and Sport Sciences (PhaNuSpo), University of Vienna, 1090 Vienna, Austria

[3] Institute of Genetics and Molecular and Cellular Biology, University of Strasbourg, CNRS UMR7104, INSERM U 1258, Illkirch-Graffenstaden, 67404, France

[4] Department of Pharmaceutical Sciences, Division of Pharmaceutical Chemistry, Faculty of Life Sciences, University of Vienna, Josef-Holaubek-Platz 2, 1090 Vienna, Austria

[5] Department of Physics, Sao Paulo State University, Rua Cristóvão Colombo 2265, São José do Rio Preto, CEP 15054-000, Brazil

[6] Institute of Physical and Theoretical Chemistry, Technische Universität Braunschweig, Gaußstraße 17, 38106 Braunschweig, Germany

[*] To whom correspondence should be addressed. Email: ya.chen@univie.ac.at

Contents of Supporting Information

Figure S1. Resazurin conversion assays in HEK293 and EL-4 mRORγt cells S3

Figure S2. Boxplot comparing RLU values of Gal4-RORγ variants S3

Figure S3. Experimental evaluation of SR2211 on Gal4-RORγ mutants S4

Figure S4. RT-qPCR analysis of compounds in EL-4-mRORγt cells S5

Figure S5. Flow cytometric analysis of EL-4-mRORγt cells S6

Table S1. Compounds selected from virtual screening for experimental evaluation S7

Table S2. Overview of plasmids and sources S8

Table S3. List of primers used in this work S9

Table S4. PCR settings for site-directed mutagenesis S10

Table S5. PCR settings for amplification of murine RORγt and addition of NheI and XhoI restriction sites S10

Table S6. Overview of nanoDSF replicates S10

Table S7. RT-qPCR measurement settings on LightCycler 480 S11

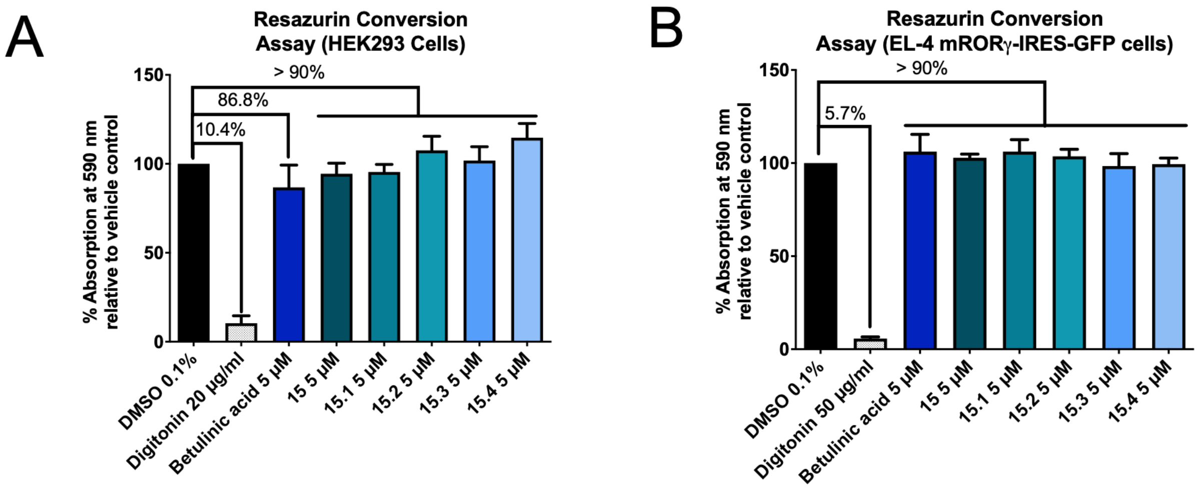

Figure S1. Resazurin conversion assays in HEK293 and EL-4 mRORγt cells. **(A)** Betulinic acid, **15**, and the follow-up derivatives show no cytotoxic effects in HEK293 cells as determined by a resazurin conversion assay. The assay was performed by treating the cells for 18 h with either the vehicle control, the positive control for cytotoxicity (digitonin at 20 µg/ml) or the compounds of interest at 5 µM. After adding resazurin (10 µg/ml), cells were incubated for another 5 h before RFU values were measured at λem = 590 nm. Data are expressed as % absorption at 590 nm relative to the vehicle control (set to 100%) and presented as mean ± SD of at least three biological replicates (n≥3), measured in technical quadruplicates. **(B)** Betulinic acid, **15**, and the follow-up derivatives show no cytotoxic effects in EL-4-mRORγt cells as determined by a resazurin conversion assay. The assay was performed by treating the cells for 20-24 h with either the vehicle control, the positive control for cytotoxicity (digitonin at 50 µg/ml) or the compounds of interest at 5 µM. After adding resazurin (10 µg/ml), cells were incubated for another 5 h before RFU values were measured at λem = 590 nm. Data are expressed as % absorption at 590 nm relative to the vehicle control (set to 100%) and presented as mean ± SD of at least three biological replicates (n≥3), measured in technical quadruplicates.

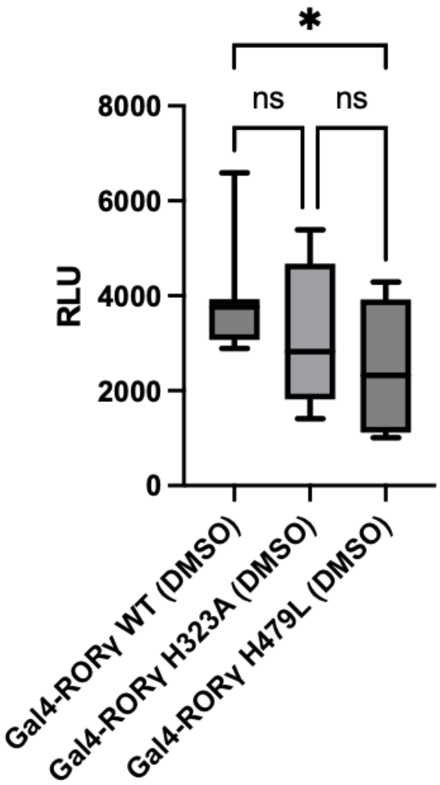

Figure S2. Boxplot (min to max, line = median) comparing RLU values of Gal4-RORγ WT, Gal4-RORγ H323A, and Gal4-RORγ H479L. RLU values were taken from three biological replicates (n = 3) measured in technical quadruplicates. One-way ANOVA, followed by Tukey's post hoc test, was used for statistical analysis. **** $p \leq 0.0001$, *** $p \leq 0.001$, ** $p \leq 0.01$, * $p \leq 0.05$, ns $p >$ 0.05.

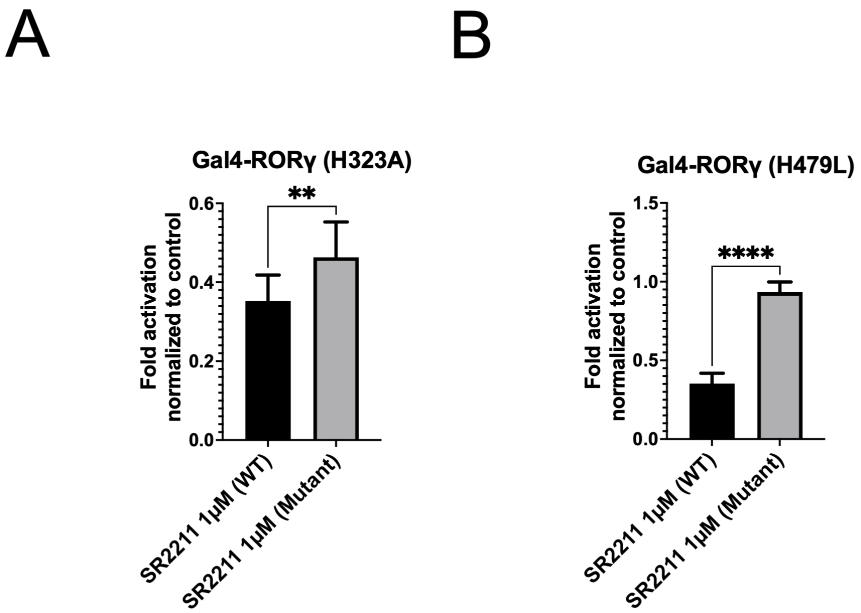

Figure S3. Experimental evaluation of SR2211 on the Gal4-RORγ H323A mutant **(A)** and the H479L mutant **(B)**. SR2211 was tested at 1 µM concentration in a cell-based Gal4-RORγ luciferase assay, where the mutations were introduced into the RORγ-LBD through site-directed mutagenesis. The luminescence signals derived from the luciferase reporter were normalized to eGFP fluorescence and expressed as fold activation normalized to the vehicle control. Bar charts represent transactivation activities as mean ± SD of three biological replicates (n = 3) measured in technical quadruplicates. An unpaired two-tailed t-test was used for statistical analysis. **** $p \leq 0.0001$, *** $p \leq 0.001$, ** $p \leq 0.01$, * $p \leq 0.05$, no indication $p > 0.05$ (activity of SR2211 on WT vs. mutant receptor).

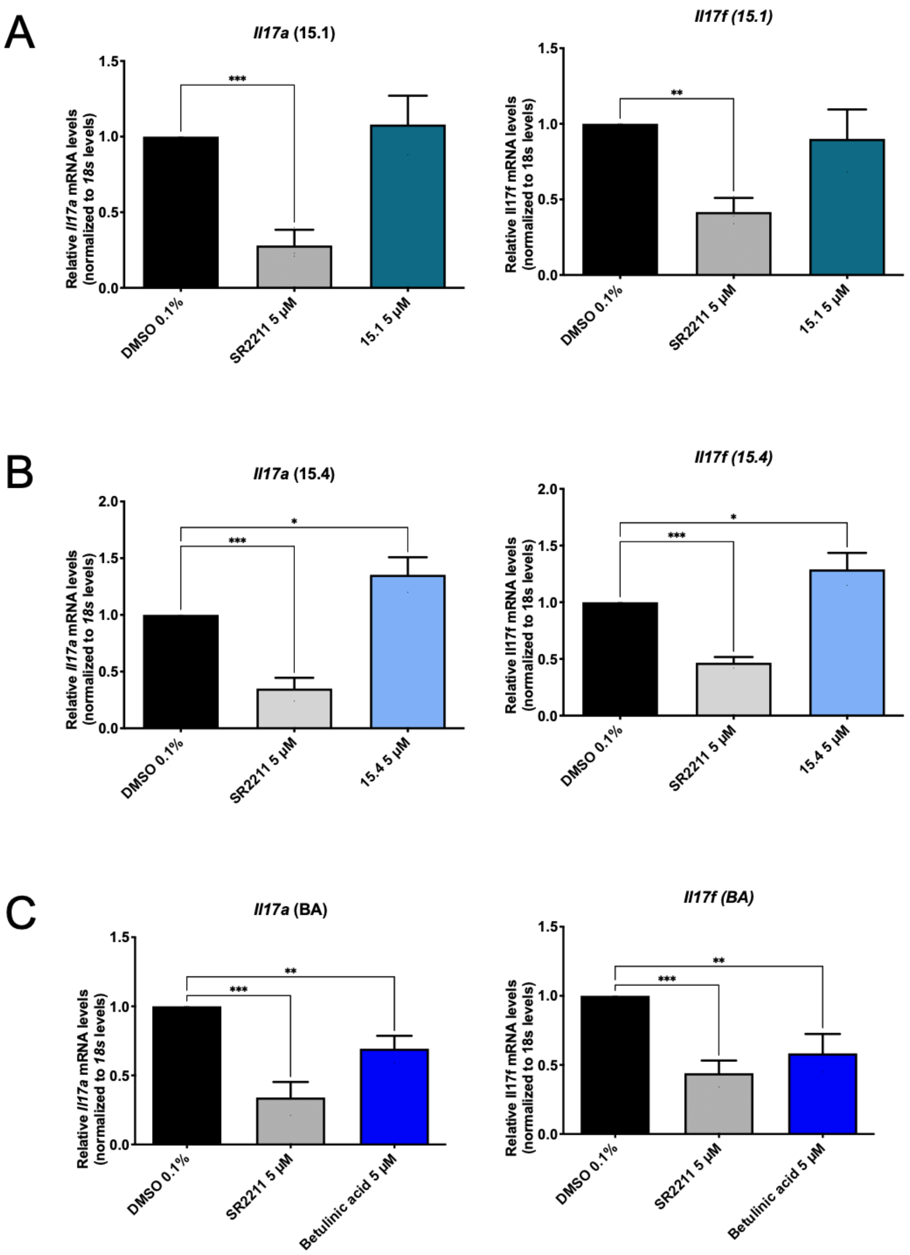

Figure S4. RT-qPCR analysis of **15.1**, **15.4**, and betulinic acid in EL-4-mRORγt cells. **(A-C)** Analysis of *Il17a/f* gene expression levels in EL-4-mRORγt cells pretreated with vehicle control, the positive control SR2211 (5 μM), or the compounds of interest at the indicated concentrations for 20-24 hours. Treatment was followed by a 4.5-hour stimulation with PMA and ionomycin, after which total RNA was isolated, reversely transcribed into cDNA, and subjected to RT-qPCR. Data are presented as mean ± SD from three biological replicates (n=3) performed in technical duplicates or triplicates. One-way ANOVA, followed by Dunnett's post hoc test, was used for statistical analysis. **** $p \leq 0.0001$, *** $p \leq 0.001$, ** $p \leq 0.01$, * $p \leq 0.05$, no indication $p > 0.05$ as compared to vehicle control.

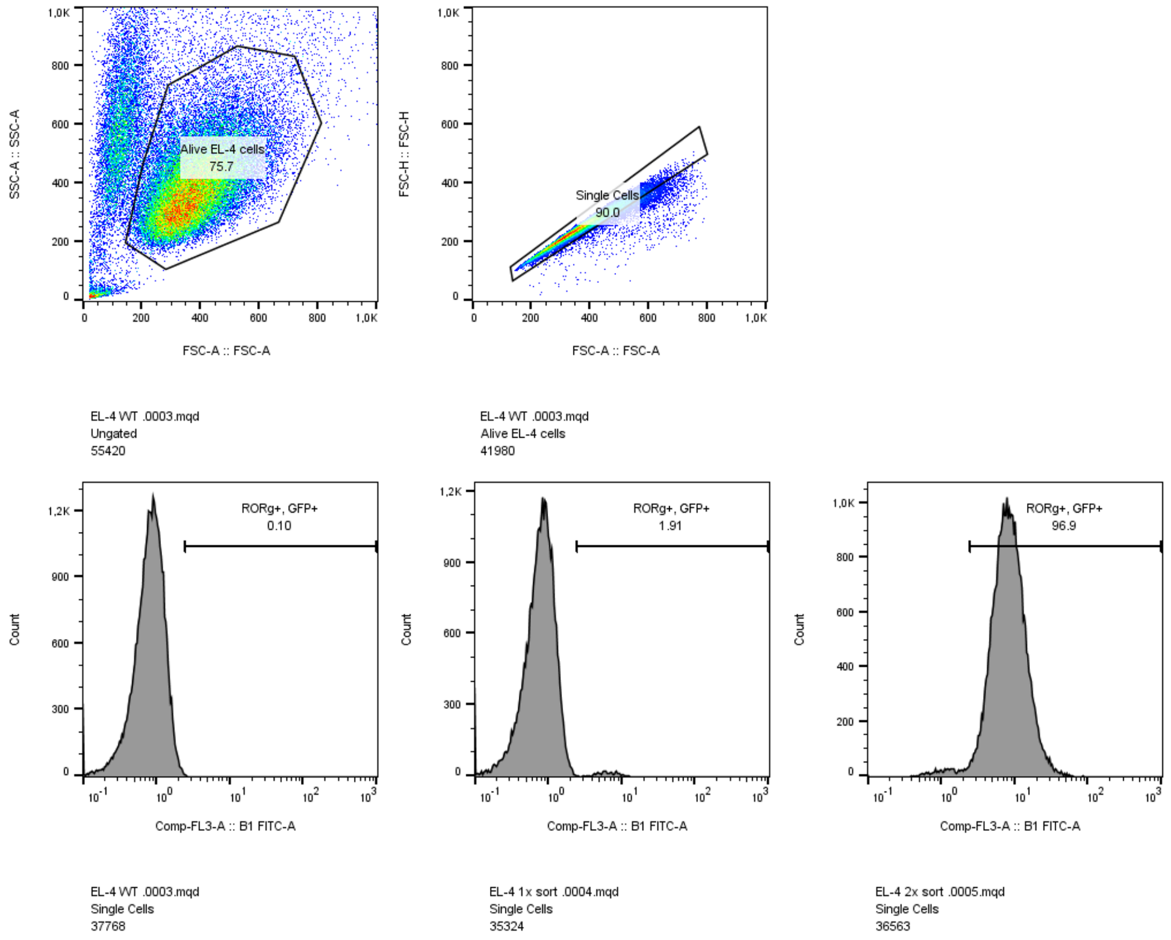

Figure S5. Flow cytometric analysis of EL-4-mRORγt cells. EL-4 WT, EL-4-mRORγt (1x sorted), and EL-4-mRORγt (2x sorted) were measured on a MACSQuant Analyzer 10 flow cytometer. The upper panel shows the gating strategy for EL-4 cells (left) and the doublet exclusion (right), while the lower panel shows the percentage of mRORγt+-GFP+ cells in the WT (left), 1x sorted (middle), and 2x sorted (right) populations. Aliquots of the 2x sorted population were frozen and used for RT-qPCR experiments.

Table S1. Compounds selected from virtual screening for experimental evaluation.

ID	MolPort ID	Supplier ID	SMILES	Picked by docking with PDB structure	Glide docking score (kcal/mol)
**1**	MolPort-029-885-220	Y042-6511	CSCC[C@@H]1NC(=O)c2cc(NC(=O)CCn3cnc4ccccc4c3=O)ccc2NC1=O	4WQP	-9.8
**2**	MolPort-035-692-421	C073-4674	Cc1ccc(NC(=O)C2[C@H]3OC4(C=C3)[C@@H]2C(=O)N(Cc2ccccc2F)C4C(=O)NC2CCCCC2)cc1Cl	5NU1	-12.5
**3**	MolPort-002-526-467	STL522502	Fc1ccc(CCNC(=O)c2cc3c4ccccc4[nH]c3c(CCc3ccccc3)n2)cc1	5NU1	-12.3
**4**	MolPort-002-513-581	STK996882	COc1ccc(cc1CSc1nc2ccccc2[nH]1)C1NCCc2c1[nH]c1ccccc21	4WQP	-9.8
**5**	MolPort-001-990-359	STK099349	COc1ccc(CC(=O)Nc2cc3oc4ccccc4c3cc2OC)cc1	5NU1	-11.8
**6**	MolPort-039-055-902	STL476697	COCCn1cnc2ccc(NC(=O)Cc3c(C)c4ccc(O)cc4oc3=O)cc2c1=O	5NU1	-11.8
**7**	MolPort-002-534-062	STL532829	CC(C)C[C@H](NC(=O)Cn1c(=O)[nH]c2ccccc2c1=O)C(=O)N[C@@H](CCCNC(N)=O)C(O)=O	6J3N	-11.5
**8**	MolPort-006-822-083	STK642752	COc1ccc2[nH]c3CCN(Cc3c2c1)C(=O)C1c2ccccc2Oc2ccccc12	4WQP	-10.2
**9**	MolPort-044-179-131	STL536931	Cc1c(OCc2ccc(F)cc2F)ccc2c(C)c(CCC(=O)N3CCC4(O)CCCCC4C3)c(=O)oc12	6J3N	-11.4
**10**	MolPort-000-841-294	STL517425	Cl.COc1ccc(cc1)-c1cc(=O)oc2c(CNCc3ccc(OC)c(OC)c3)c(O)ccc12	4WQP	-9.9
**11**	MolPort-035-701-318	STL496064	O=C(CCCCCN1C2N(C(=O)c3ccccc23)c2ccccc2C1=O)Nc1cccnc1	5NTP	-12.0
**12**	MolPort-002-531-927	STL529732	Cc1oc2cc3oc(=O)c(CC(=O)NCC(O)c4ccccc4)c(C)c3cc2c1C	4WQP	-9.7
**13**	MolPort-009-759-232	STL536614	O=C(CCc1ccccc1)Nc1ccc(nc1)N1C[C@H]2C[C@@H](C1)c1cccc(=O)n1C2	5NU1	-12.4
**14**	MolPort-004-858-727	STL465232	OC[C@H](Cc1ccccc1)NC(=O)COc1ccc(cc1)-c1cc2ccccc2oc1=O	4WQP	-9.9
**15**	MolPort-007-980-909	STL526289	CC(=C)[C@@H]1CCC2(CC[C@]3(C)C(CCC4[C@@]5(C)C\C(=C\c6ccco6)C(=O)C(C)(C)C5CC[C@@]34C)C12)C(O)=O	6J3N	-12.5
**16**	MolPort-000-854-552	STL034255	NC(=O)C1CCN(CCOc2ccc3c(c2)occ(-c2ccccc2)c3=O)CC1	4WQP	-10.3
**17**	MolPort-000-855-397	STL535026	COc1ccc(CNC(=O)CC[C@@H]2NC(=O)N(CC3(CCOCC3)c3ccc(OC)cc3)C2=O)cc1	5NU1	-11.8
**18**	MolPort-002-515-907	STL527184	Fc1c(F)c(F)c(Oc2ccc(cc2)N2C(=O)N[C@@H](Cc3c[nH]c4ccccc34)C2=O)c(F)c1F	4WQP	-10.1
**19**	MolPort-035-873-904	STL522764	COc1ccc(CC(=O)Nc2ccc3ncn(CCC(C)C)c(=O)c3c2)cc1	5NU1	-11.6
**20**	MolPort-000-843-680	STL535149	CC(C)C[C@@H](CO)NC(=O)COc1ccc2c(c1)occ(-c1ccccc1)c2=O	4WQP	-9.7
**21**	MolPort-000-918-127	STK860045	COc1ccc(CC(=O)Nc2oc(c(c2C#N)-c2ccccc2)-c2ccccc2)cc1	5NU1	-12.7
**22**	MolPort-002-533-067	STL565695	Cc1cc(=O)oc2c3CCC(C)(C)Oc3cc(OCC(=O)NCCCN3CCCC3=O)c12	5NU1	-11.6
**23**	MolPort-002-666-473	STK612635	COc1ccc(cc1)-c1cc(=O)oc2cc(OCC(=O)Nc3cccnc3)ccc12	5NTP	-11.2

Table S2. Overview of plasmids and sources.

Plasmid (backbone)	Function	Antibiotic resistance	Provider
Gal4-hRORγ (pBIND)	Encodes for a hRORγ-LBD:Gal4-DBD fusion protein; Gal4 binds to upstream activating sequences (UAS).	Zeocin	Prof. Laura A. Solt (UF Scripps Biomedical Research, University of Florida, Jupiter, FL, USA)
pTK-MH100x4-LUC (TK-LUC)	Encodes for a luciferase reporter under the control of UAS.	Ampicillin	Prof. Ronald Evans (Salk Institute for Biological Studies, La Jolla, CA, USA)
Full-length human RORγ (pCMV-Sport 6)	Encodes for the full-length human RORγ.	Ampicillin	Prof. Patrick R. Griffin (UF Scripps Biomedical Research, University of Florida, Jupiter, FL, USA)
RORE-LUC (pGL3)	Encodes for a luciferase reporter under the control of RORE.	Ampicillin	Prof. Patrick R. Griffin (UF Scripps Biomedical Research, University of Florida, Jupiter, FL, USA)
Full-length murine RORγt (pcDNA3.1)	Encodes for the full-length murine RORγt.	Ampicillin	Prof. Laura A. Solt (UF Scripps Biomedical Research, University of Florida, Jupiter, FL, USA)
hROR 264-518 (pET15b)	Encodes for the ligand binding domain of human RORγ	Ampicillin	

Table S3. List of primers used in this work.

Primer name	Forward primer (5’-3’)	Reverse primer (5’-3’)	Comments
hRORg-Gal4 H323A	ACG GTG TGC CCA CGC CCT CAC CGA GGC C	GGC CTC GGT GAG GGC GTG GGC ACA CCG T	Mutagenic primer used to generate the Gal4-hRORγ H323A mutant
hRORg-Gal4 H479L	CCT GTG TAG CCA GCT TGT GGA AAG GCT GC	GCA GCC TTT CCA CAA GCT GGC TAC ACA GG	Mutagenic primer used to generate the Gal4-hRORγ H479L mutant
GAL4-BD	TCA TCG GAA GAG AGT AG	/	Standard sequencing primer from Microsynth
RORg-Gal4 LBD sequencing fwd 1	CTC CCT GAC AGA GAT AGA GC	/	Self-designed sequencing primer
mRORgt pIRES2-eGFP NheI fwd / XhoI rev	AAA GCT AGC GGT GGA ATA CCA TGA GAA CAC	AAA CTC GAG TCG AGT CAC TTT GAC AGC CCC	Primers for PCR-amplification of mRORγt and addition of NheI and XhoI restriction sites
IRES-for	TAG GCG TGT ACG GTG GG	/	Standard sequencing primer from Microsynth
mil17a	CAA CCG TTC CAC GTC ACC C	GAG CTT CCC AGA TCA CAG AGG G	RT-qPCR primer
mil17f	GAA ACC AGC ATG AAG TGC ACC C	TGC TAC CTC CCT CAG AAT GGC	RT-qPCR primer
mil23r	AGG CTT TTC GGA ACC TCA TGC	GTC AGA TTG CTG GGG GCA TC	RT-qPCR primer
m18s	GTA ACC CGT TGA ACC CCA TT	CCA TCC AAT CGG TAG TAG CG	RT-qPCR primer

Table S4. PCR settings for site-directed mutagenesis.

Cycle step	Temperature	Time	Cycles
Initial denaturation	95 °C	2 minutes	1
Denaturation	95 °C	15 seconds	18
Primer annealing	60 °C	10 seconds	
Extension	68 °C	3 minutes (30 seconds/kb of plasmid length)	
Final synthesis	68 °C	5 minutes	1
Cooling	4 °C		1

Table S5. PCR settings for amplification of murine RORγt and addition of NheI and XhoI restriction sites.

Cycle step	Temperature	Time	Cycles
Initial denaturation	95 °C	4 minutes	1
Denaturation	95 °C	30 seconds	29
Primer annealing	60 °C	30 seconds	
Extension	72 °C	1 minute (30 seconds/kb of plasmid length)	
Final synthesis	72 °C	3 minutes	1
Cooling	4 °C		1

Table S6. Overview of nanoscale Differential Scanning Fluorimetry replicates.

	**First biological replicate**			**Second biological replicate**		
**Sample id**	**Tm (°C)** technical replicate 1	**Tm (°C)** technical replicate 2	**Tm (°C)** technical replicate 3	**Tm (°C)** technical replicate 1	**Tm (°C)** technical replicate 2	**Tm (°C)** technical replicate 3
Vehicle control	41.5	41.4	41.4	41.5	41.8	41.8
T0901317	51.6	52.1	52.0	52.0	52.0	52.1
Betulinic acid	50.5	50.6	50.8	50.4	50.9	50.6
**15**	50.9	50.8	50.8	51.6	51.3	51.6
**15.1**	47.5	47.5	48.1	47.3	47.2	47.1
**15.2**	43.3	43.3	43.4	44.1	44.3	44.1
**15.3**	44.5	44.7	44.2	44.1	44.1	44.3
**15.4**	42.6	42.7	42.9	42.5	42.6	42.5

Table S7. RT-qPCR measurement settings on LightCycler 480.

Cycle step	Temperature	Time	Cycles
Initial denaturation	95 °C	60 seconds	1
Denaturation	95 °C	15 seconds	45
Primer annealing/extension	60 °C	30 seconds (+ plate read)	
Melting curve	55-95 °C		1
Cooling	40 °C		1
